# Reduced Susceptibility of *Plasmodium falciparum* to Artesunate in Southern Myanmar

**DOI:** 10.1371/journal.pone.0057689

**Published:** 2013-03-08

**Authors:** Myat P. Kyaw, Myat H. Nyunt, Khin Chit, Moe M. Aye, Kyin H. Aye, Moe M. Aye, Niklas Lindegardh, Joel Tarning, Mallika Imwong, Christopher G. Jacob, Charlotte Rasmussen, Jamie Perin, Pascal Ringwald, Myaing M. Nyunt

**Affiliations:** 1 Department of Medical Research (Lower Myanmar), Yangon, The Republic of the Union of Myanmar; 2 Mahidol-Oxford Tropical Medicine Research Unit, Mahidol University, Bangkok, Thailand; 3 Center for Tropical Medicine, Nuffield Department of Clinical Medicine, University of Oxford, Oxford, United Kingdom; 4 Department of Molecular Tropical Medicine and Genetics, Mahidol University, Bangkok, Thailand; 5 Malaria Group, Howard Hughes Medical Institute/Center for Vaccine Development, University of Maryland School of Medicine, Baltimore, Maryland, United States of America; 6 Drug Resistance and Containment Unit, Global Malaria Programme, World Health Organization, Geneva, Switzerland; 7 Department of International Health, Johns Hopkins University Bloomberg School of Public Health, Baltimore, Maryland, United States of America; 8 Division of Clinical Pharmacology, Johns Hopkins University School of Medicine, Baltimore, Maryland, United States of America; Kenya Medical Research Institute - Wellcome Trust Research Programme, Kenya

## Abstract

**Background:**

*Plasmodium falciparum* resistance to artemisinins, the first line treatment for malaria worldwide, has been reported in western Cambodia. Resistance is characterized by significantly delayed clearance of parasites following artemisinin treatment. Artemisinin resistance has not previously been reported in Myanmar, which has the highest falciparum malaria burden among Southeast Asian countries.

**Methods:**

A non-randomized, single-arm, open-label clinical trial of artesunate monotherapy (4 mg/kg daily for seven days) was conducted in adults with acute blood-smear positive *P. falciparum* malaria in Kawthaung, southern Myanmar. Parasite density was measured every 12 hours until two consecutive negative smears were obtained. Participants were followed weekly at the study clinic for three additional weeks. Co-primary endpoints included parasite clearance time (the time required for complete clearance of initial parasitemia), parasite clearance half-life (the time required for parasitemia to decrease by 50% based on the linear portion of the parasite clearance slope), and detectable parasitemia 72 hours after commencement of artesunate treatment. Drug pharmacokinetics were measured to rule out delayed clearance due to suboptimal drug levels.

**Results:**

The median (range) parasite clearance half-life and time were 4.8 (2.1–9.7) and 60 (24–96) hours, respectively. The frequency distributions of parasite clearance half-life and time were bimodal, with very slow parasite clearance characteristic of the slowest-clearing Cambodian parasites (half-life longer than 6.2 hours) in approximately 1/3 of infections. Fourteen of 52 participants (26.9%) had a measurable parasitemia 72 hours after initiating artesunate treatment. Parasite clearance was not associated with drug pharmacokinetics.

**Conclusions:**

A subset of *P. falciparum* infections in southern Myanmar displayed markedly delayed clearance following artemisinin treatment, suggesting either emergence of artemisinin resistance in southern Myanmar or spread to this location from its site of origin in western Cambodia. Resistance containment efforts are underway in Myanmar.

**Trial Registration:**

Australian New Zealand Clinical Trials Registry ACTRN12610000896077

## Introduction

Artemisinin-based combination therapies (ACTs) are the first-line treatment for *Plasmodium falciparum* malaria worldwide because of their high clinical efficacy, rapid parasite clearance and fast clinical recovery. ACT efficacy is now threatened by the recent confirmation of *P. falciparum* resistance to artemisinins in western Cambodia [1]–[3]. Despite efforts to contain artemisinin resistance in western Cambodia, concerns have arisen [4] that resistant parasites from Cambodia may have spread to, or alternatively arisen independently in, other countries in the Greater Mekong Sub-Region of Southeast Asia, including Myanmar [5].

Artemisinin-resistant *P. falciparum* is defined clinically as delayed parasite clearance following artesunate monotherapy. No molecular marker of artemisinin resistance has been validated, and correlation between *in vivo* clinical outcomes and *in vitro* measures of parasite susceptibility is poor [2]. Presently only the clinical definition can be used to confirm resistance. A rapid initial reduction in parasite density is a life-saving feature that distinguishes artemisinins form other antimalarial drugs and that reduces the mortality of severe malaria [6], [7]. Elimination of microscopically detectable parasitemia by 24–48 hours, is typical of *P. falciparum* that is fully susceptible to artemisinins [8]–[11] and persistent parasitemia 72 hours after commencing ACT treatment is interpreted as evidence of possible resistance [12]. The rate of parasite clearance (reported as parasite clearance half-life), and parasite clearance time in hours (controlling for the starting parasitemia), are considered the definitive measures of parasite susceptibility or resistance to artemisinins [13], [14].

A World Health Organization (WHO)-led surveillance program to monitor ACT efficacy was initiated in 2006 in Myanmar. Progressive increases in the proportion of patients with persistent parasitemia 72 hours after starting treatment were observed in Myanmar starting in 2009 [15], particularly in the border areas near Thailand. Similarly a progressive decline in the rate of parasite clearance from 2001 to 2010 was reported from a study conducted on the north-western border of Thailand with Myanmar in patients with hyperparasitemia who were treated with various artesunate-based combination therapies [16].To confirm these observations, we conducted a prospective, open-label clinical study to measure parasite clearance, efficacy and pharmacokinetics of curative artesunate monotherapy. Artesunate monotherapy was used to assess the independent ability of artemisinins to clear *P. falciparum* without confounding by the effect of a partner drug.

## Methods

The protocol for this trial and supporting CONSORT checklist are available as supporting information; see Checklist S1 and Protocol S1.

### Study Design

This was a non-randomized, single-arm, open-label clinical trial to assess parasite clearance rate following directly-observed 7-day oral artesunate (4 mg/kg) daily monotherapy for the treatment of adult participants with uncomplicated blood smear positive P. falciparum malaria. The study was approved by ethical review committees of the Department of Medical Research (Lower Myanmar), the Myanmar Ministry of Health, and the WHO.

### Study Site and Participants

The study was conducted in Palm Tree Hospital in Kawthaung, a town located at southern tip of Myanmar bordering Thailand (Fig. S1). This study site was selected since it was one of the areas of Myanmar with the highest annual incidence rates of clinical malaria (5–49 per 1,000) [17] and a high index of suspicion of artemisinin resistance. Rural residents of a heavily forested area along the coastal plain and river valleys were enrolled in March and April, 2011. Inclusion criteria included age 18–55 years, mono-infection with *P. falciparum*, asexual parasite density 10,000–100,000/µl, fever defined as axillary temperature >37.5°C or history of subjective fever in the last 24 hours, ability to tolerate oral intake, provision of written informed consent, and agreement to comply with the study protocol, including hospitalization for seven days. Exclusion criteria included severe malaria [18], severe malnutrition, pregnancy, lactation, mixed Plasmodia infection, clinical evidence of infection other than malaria, history of chronic medical illness, splenectomy, hypersensitivity to artesunate or related compounds, or reported use of drugs with antimalarial activity within 48 hours before enrollment.

### Study Procedures

Once-daily oral doses of artesunate 4 mg/kg/day, procured by WHO from Guilin Pharmaceutical Co. Ltd. (Shanghai, China; lot number AS091001; expiration date October 2011) were administered under direct observation with 8 oz. of milk (to standardize diet before drug administration which may impact drug absorption) on days 0–6. Treatment was repeated in case of vomiting within 30–60 minutes after drug administration. Parasite density was assessed by blood smear every 12 hours by two independent microscopists, using the average of the two readings, until two consecutive negative smears were obtained. The exact time of artesunate administration, and the scheduled and actual time of blood collection for smear examination were recorded for each participant. At each finger-prick for blood smear, dried blood spots were also collected on Whatman 3 MM filter paper. Filter papers were air-dried, labeled and stored in individual plastic bags with desiccant until analyzed.

Participants were evaluated daily for solicited and unsolicited adverse events. Blood was collected for pharmacokinetic analysis of artesunate and its major metabolite dihydroartemisinin (DHA) in pre-chilled blood collection tubes containing fluoride-potassium oxalate, immediately before, and 0.25, 0.5, 0.75, 1.0, 1.25, 1.5, 3, 4, 6 and 8 hours after the first dose of artesunate. Within 15 minutes of collection, blood was centrifuged at 4°C at 2000×g for 7 minutes, and plasma was collected and stored in liquid nitrogen until analysis.

Participants were discharged from the hospital on day 7 and monitored as outpatients on days 14, 21 and 28. Hemoglobin was measured on days 14 and 28. Finger-prick blood was collected for malaria smear and filter paper blood spots on days 7, 14, 21 and at any time the participant felt unwell.

Treatment outcomes were classified following WHO-recommended methods for monitoring antimalarial drug efficacy [19] as early treatment failure (severe malaria or lack of adequate response to drug treatment on days 0–3), late clinical failure (severe malaria or lack of adequate response to drug treatment on days 4–28), late parasitological failure (parasitemia without clinical symptoms on days 7–28, without meeting criteria for early treatment or late clinical failure), or adequate clinical and parasitological response (absence of parasitemia on day 28, without meeting any failure criteria). Smear-proven recurrent *P. falciparum* or *P. vivax* was treated, following the National Malaria Treatment Guidelines, with unsupervised standard WHO-recommended regimens of co-formulated artemether-lumefantrine (the standard ACT under the National Malaria Treatment Policy in Myanmar) 80/480 mg every 12 hour for three days or chloroquine 25 mg base/kg once a day for three days followed by primaquine 0.25 mg/kg once a day for 14 days, respectively.

### Laboratory Methods

Microscopy: Giemsa-stained thick and thin blood smears were prepared for parasite density determination and speciation, respectively. Parasites were counted against white blood cells (WBC) and parasite density per µl was calculated by dividing the number of asexual parasites by the number of WBC counted (200, or 500 if the parasite density was less than 10/µl) and multiplying by an assumed WBC count of 6,000/µl. Smears were considered negative when no asexual parasites were found after counting 1,000 WBC. Molecular analysis of recurrent infections: DNA was extracted from dried blood spots following manufacturer’s instructions (QIAamp DNA Mini Kit, Hilden, Germany), and amplified and analyzed as previously described to distinguish whether post-treatment recurrent infections were new infections or recrudescences [20]. Paired pre- and post-treatment samples were assessed in triplicate, comparing sizes of fragments of merozoite surface protein −1 and −2 and glutamate-rich protein genes. *P. vivax* infection was identified by nested PCR of 18ssrRNA using previously described methods [21].

Pharmacokinetic analysis: Plasma concentrations of artesunate and DHA were measured by liquid chromatography-tandem mass spectroscopy after solid phase extraction using published validated methods [22]. The total assay coefficients of variation in quality control samples were less than 8% at each low, medium and high concentration. The lower limits of quantification were 1.2 ng/ml and 2.0 ng/ml for artesunate and DHA, respectively.

### Statistical Analysis

Parasite clearance half-life, parasite clearance time, and the proportion with persistent parasitemia at 72 hours following start of treatment were co-primary endpoints. Because prior studies only provided information on the latter endpoint, parasitemia at 72 hours was used for sample size determination. Assuming <5% 72-hour parasitemia is in areas with no resistance [9], a minimum sample size of 53 was required to identify at least 15% of participants with persistent parasitemia at 72 hours (with 5% one-sided type I error and 80% power). Using published data that recently became available, we assumed that the mean parasite clearance half-life of sensitive parasites was 4.4 hours (95% confidence interval 0.6–15.8 hours), approximated from the observation of the time required for a 50% reduction of initial parasitemia from a Laotian population where there was no evidence of artemisinin resistance [11]. With the sample size of 53 and one-sided type I error of 5%, we had more than 90% statistical power to identify the mean of the population clearance half-life 6.2 hours or longer, if the parasite population in the study were resistant to artemisinin.

Data were double-entered into the Microsoft Excel database and analyzed using Stata (version 10.0; Stata, College Station, TX), and R (version 2,15,0; R foundation for statistical computing, Vienna, Austria). R and SigmaPlot (version 10.0; Systat software, San Jose, CA) were used to produce figure illustrations. Parasite clearance half-lives were estimated from serial parasite counts using the publicly available WorldWide Antimalarial Resistance Network (WWARN) Parasite Clearance Estimator [14], [23]. Parasite clearance half-life represents the time required for parasitemia to decrease by 50%, based on the linear slope of decline in parasitemia over time. The time course of parasitemia is illustrated using R by a step function corresponding to the percent of patients with positive parasite counts at each measurement time after initiating treatment over the course of the study. Least-square linear regression analysis was performed to assess potential modifiers of clearance half-life and parasite clearance time in hours, including age, gender, initial parasite density, body temperature, and pharmacokinetics of artesunate and DHA.

Pharmacokinetics of artesunate and DHA were assessed by standard non-compartmental modeling using WinNonlin Professional (version 3.3; Pharsight, Mountain View, CA), and described as maximal concentration (Cmax), time to Cmax, area under the concentration-time curve (AUC0-inf), and terminal elimination half-life. Total apparent oral clearance and apparent volume of distribution were estimated with assumed equivalent bioavailability (F) since there was no intravenous comparator. Pharmacokinetic data were skewed in distribution and summarized using medians and inter-quartile ranges. Data were also compared between participants with and without 72-hour persistent parasitemia, and between fast and slow clearers (defined as below or above slope half-life 6.2 hours based on recently published data) [16].

## Results

### Trial Profile and Adverse Events

**Figure 1:**
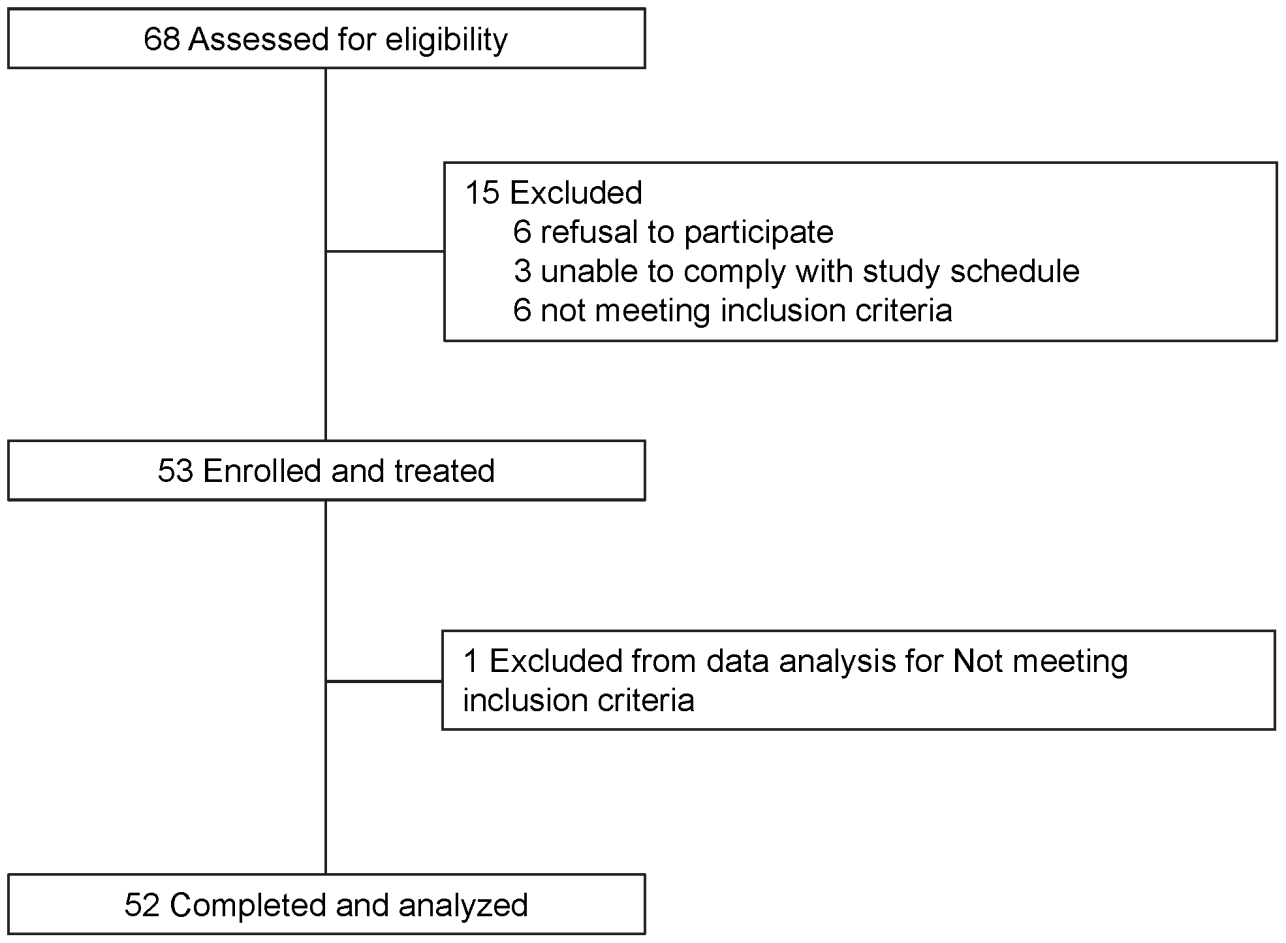
Study flow diagram.

Of a total of 68 potential candidates screened, 15 were excluded from enrollment (due to inability to comply with study protocol or evidence of mixed infections), and one from data analysis (enrollment parasitemia below the inclusion criterion); 52 were included in the final analysis (Fig. 1). Participants were young adults, >75% had fever, and >30% were anemic (hemoglobin <11 g/dl) (Table 1). All participants tolerated and complied with study procedures. No serious adverse events were reported. One participant vomited 50 min after drug administration on Day 0 and received re-treatment. At least one adverse event (headache, body aches, nausea, vomiting, fever or chill) was recorded for each participant. These adverse events were mild to moderate in severity, considered related to acute malaria illness, and resolved in 1–7 days. All participants received acetaminophen (500 mg every 8–12 hours) starting 8–12 hours after the first dose of artesunate and continuing for 2–3 days.

**Table 1:** Baseline characteristics of study participants (N = 52).

Characteristics	Value^a^
Age (year)	25.5 (21.5–39.5)
Number of female (%)	10 (19.2)
Number of smoker (%)	24 (46.2)
Weight (kg)	50.0 (46.0–53.5)
BMI (kg/m^2^)	19.2 (17.7–19.9)
Oral temperature at enrollment (°C)	38.4 (37.6–39.1)
Number of patients presenting with fever (>37.5°C) (%)	40 (76.9)
Respiration (breaths/minute)	24 (20–26)
Heart rate (beats/minute)	100 (87–106)
Blood pressure (mmHg)	113/70 (105/63–122/77)
Hemoglobin (g/dL)	12.4 (10.6–13.7)
Number of patients (%) with moderate to severe anemia^b^	11 (21.2)
Parasite density at enrollment (parasites/µL)^c^	29,952 (15,180–53,532)
Number of patients with gametocyte at enrollment (%)	0
Fever clearance time (day)	3 (2–4)

a Median (interquartile range) unless specified otherwise;

b Hemoglobin 10 g/dL or lower;

c Geometric mean (95% confidence interval).

### Treatment Outcomes and Parasite Clearance

**Figure 2:**
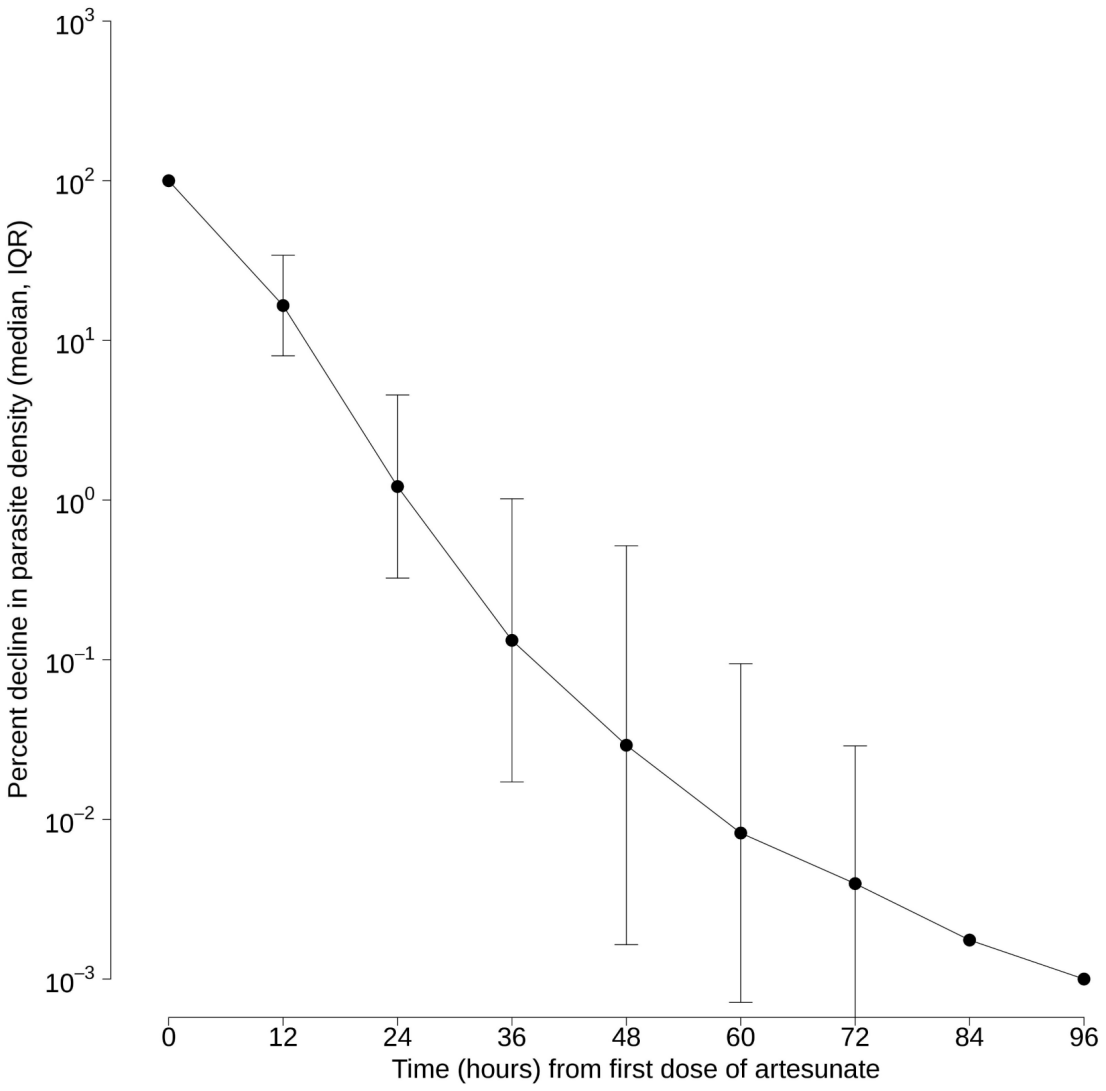
Parasite clearance curve showing linear regression of log_10_-normalized median parasite density over time.

**Figure 3:**
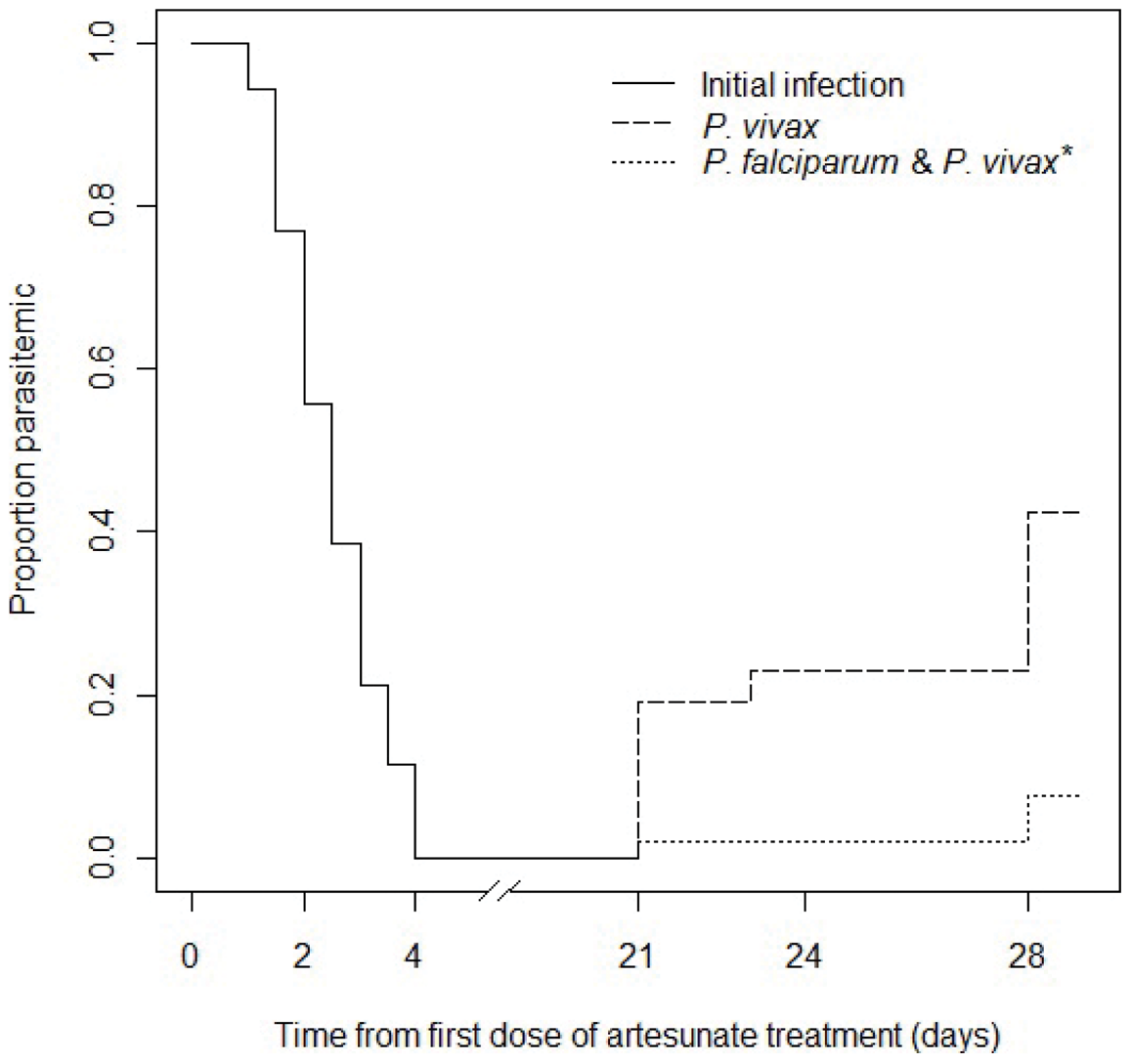
Cumulative proportion of participants developing recurrent *P. falciparum* or/and *P. vivax* infection after completion of artesunate monotherapy.

Enrollment parasite densities were moderate (geometric mean 29,952/µl; 95% confidence interval 15,180–53,532/µl). The median (range) time to fever clearance was 3 days (2–7 days) and time to parasite clearance was 60 hours (24–96 hours) (Fig. 2). Of 52 evaluable participants, 14 (26.9%) and five (9.6%) had persistent parasitemia at 72 hours and 84 hours after starting treatment, respectively (Fig. 3). A participant who vomited and was re-treated achieved complete parasite clearance in 72 hours and a 28-day adequate clinical and parasitologic response. Twenty-six participants had recurrent parasitemia on days 21–28 (Fig. 3): one *P. falciparum* on day 21; three mixed *P. falciparum* and *P. vivax* on day 28; and 22 *P. vivax* on days 21–28. All of these recurrent infections responded to treatment with artemether-lumefantrine (*P. falciparum* or mixed *P. falciparum* and *P. vivax* infection) or chloroquine and primaquine (*P. vivax* mono-infection). All recurrent *P. falciparum* cases were classified as new infections by PCR genotyping, yielding a PCR-corrected cure rate of 100%. Retrospective PCR analysis showed that at enrollment 24 of 52 (46.2%) participants were co-infected with sub-patent *P. vivax* in addition to *P. falciparum*. All but two smear-positive *P. vivax* infections found during follow up were also PCR-positive for *P. vivax*. Four additional cases of *P. vivax* were identified by PCR only.

**Figure 4:**
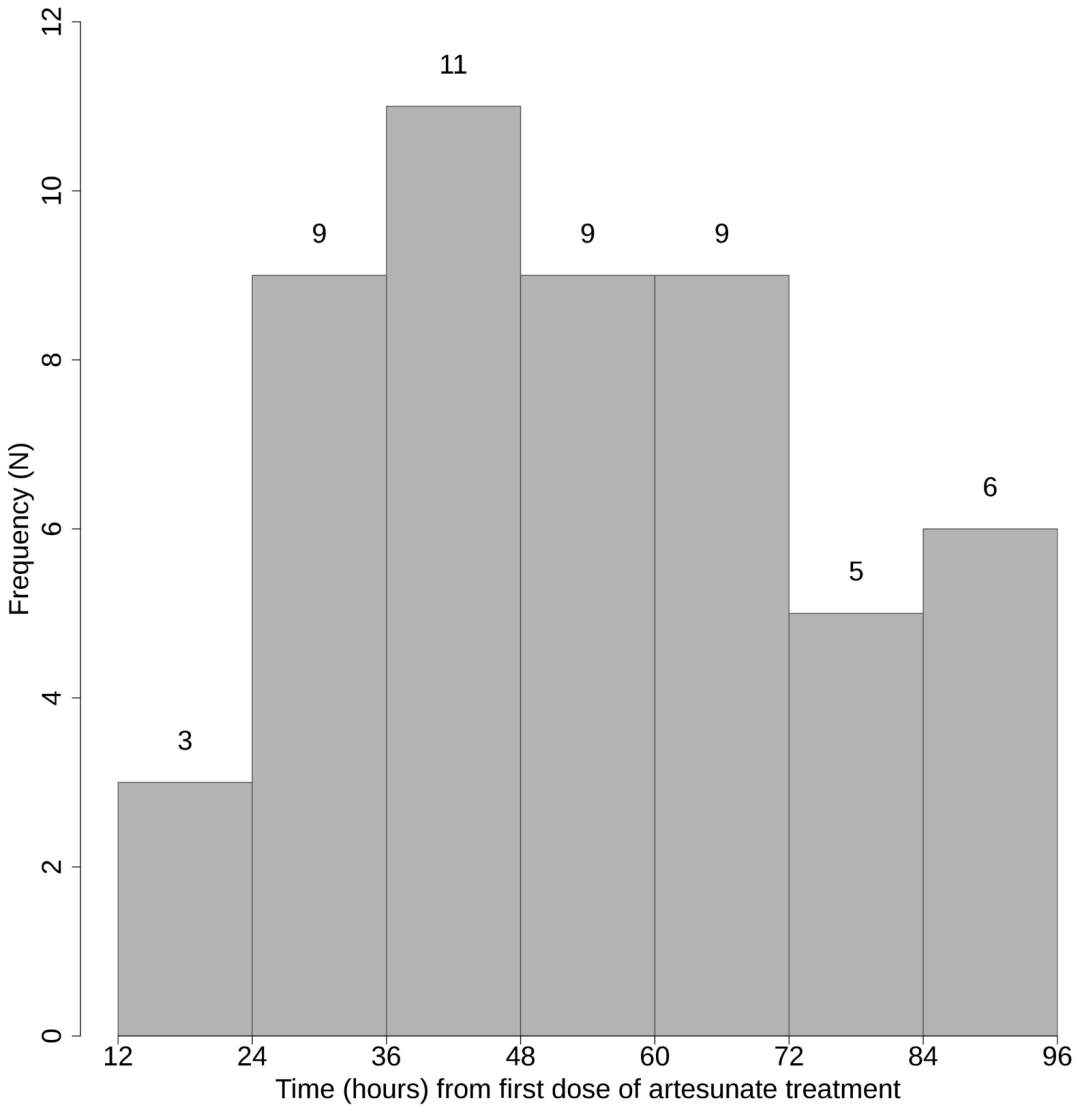
Frequency distribution of parasite clearance time.

**Figure 5:**
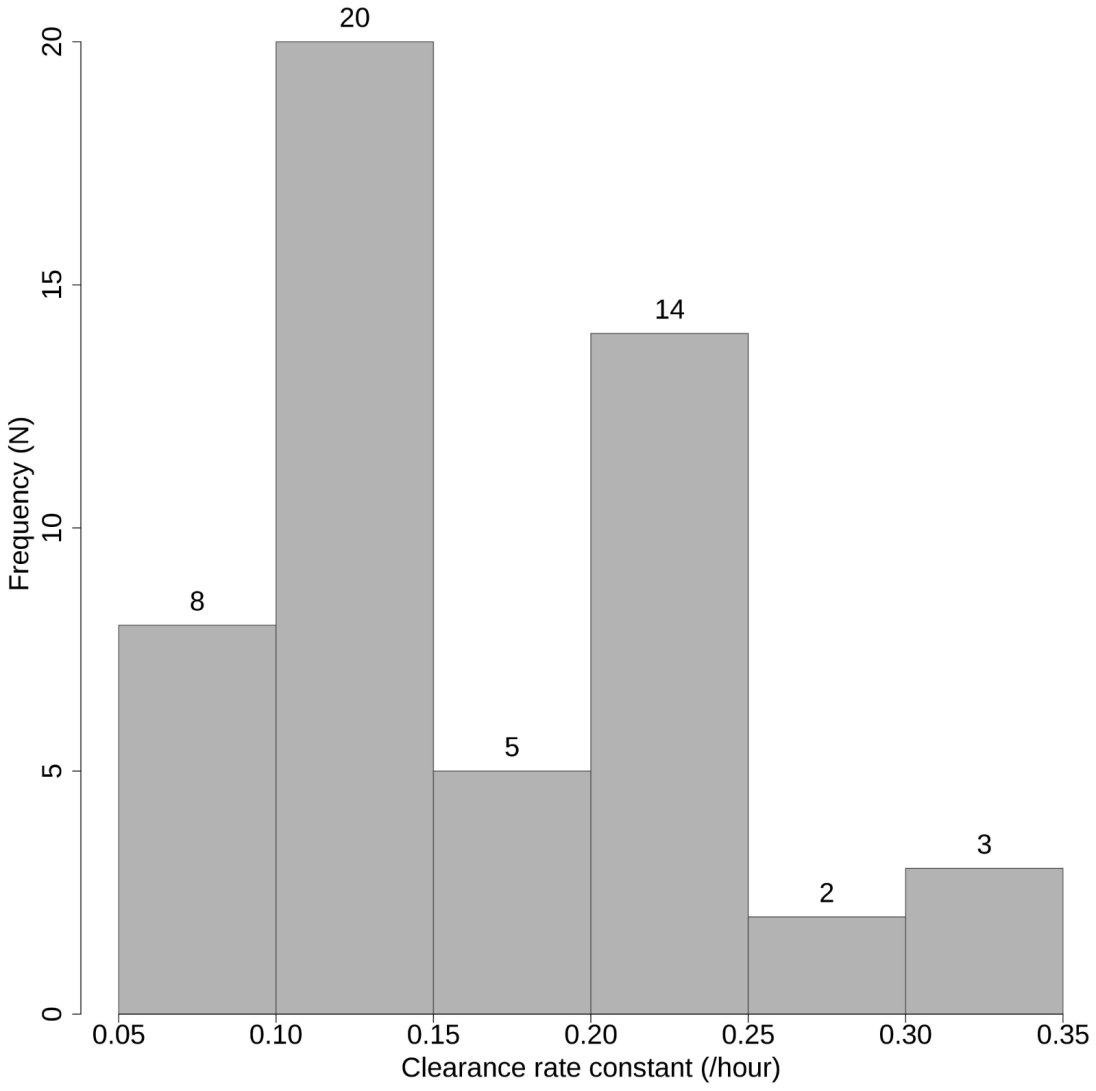
Frequency distribution of parasite clearance half-life.

**Figure 6:**
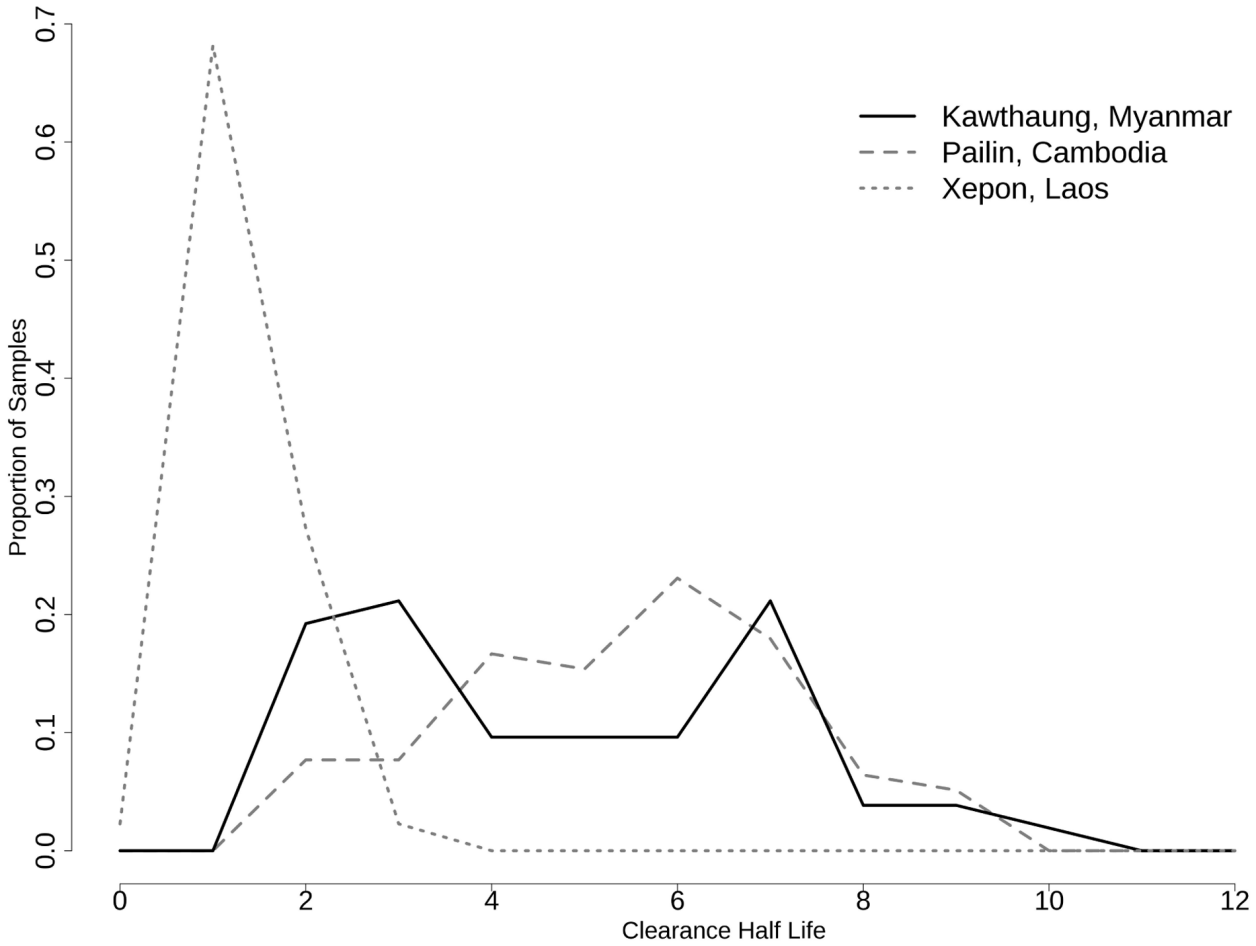
Bimodal distribution of parasite clearance half-life.

Parasite clearance curves (log-parasitemia versus time plots) were produced for each participant, and the parasite clearance half-life and the time required to reach 50%, 90% and 95% of the initial value (PC50, PC90, and PC95) are summarized in Table 2. Data points from the lag phase and tail identified in parasite clearance curves of two participants were censored to base estimation of parasite clearance half-life on the linear part of the curve [14], but all data points were included in measures of parasite clearance time. The frequency distribution of parasite clearance time and parasite clearance half-life is depicted in Fig. 4 and Fig. 5, respectively. The median (range) parasite clearance half-life was 4.8 (2.1–9.7) hours in the whole study population. The distribution of clearance half-life was bimodal (solid line in Fig. 6) with a half-life (range) of 3.0 (2.1–4.8) hours in the faster-clearing half and 6.6 (4.8–9.7) hours in the slower-clearing half. Regression analysis revealed no significant association between parasite clearance half-life and potential modifiers including age, gender, smoking, initial parasite density (Fig. S2), maximal plasma concentrations (C_max_) (Fig. S3) and total exposure (AUC) of dihydroartemisinin (Fig. S4) or artesunate (data not shown).

**Table 2:** Parasite clearance.

Parasite Clearance	Median (interquartile range) (range)
Parasite clearance time (hours)^a^	60.0 (48.3–72.6) (24.0–96.2)
Parasite clearance half-life (hours)^b^	4.8 (3.0–6.6) (2.1–9.7)
PC_50_ (hours)^c^	6.0 (4.1–9.4) (0.76–47.8)
PC_90_ (hours)^c^	15.3 (11.9–23.1) (3.7–59.2)
PC_95_ (hours)^c^	20.9 (15.0–30.1) (8.3–64.1)

a Time for complete clearance;

b Time for 50% reduction in parasite density;

c Time to 50, 90 and 95% of initial density.

### Pharmacokinetics of Artesunate and DHA

**Figure 7:**
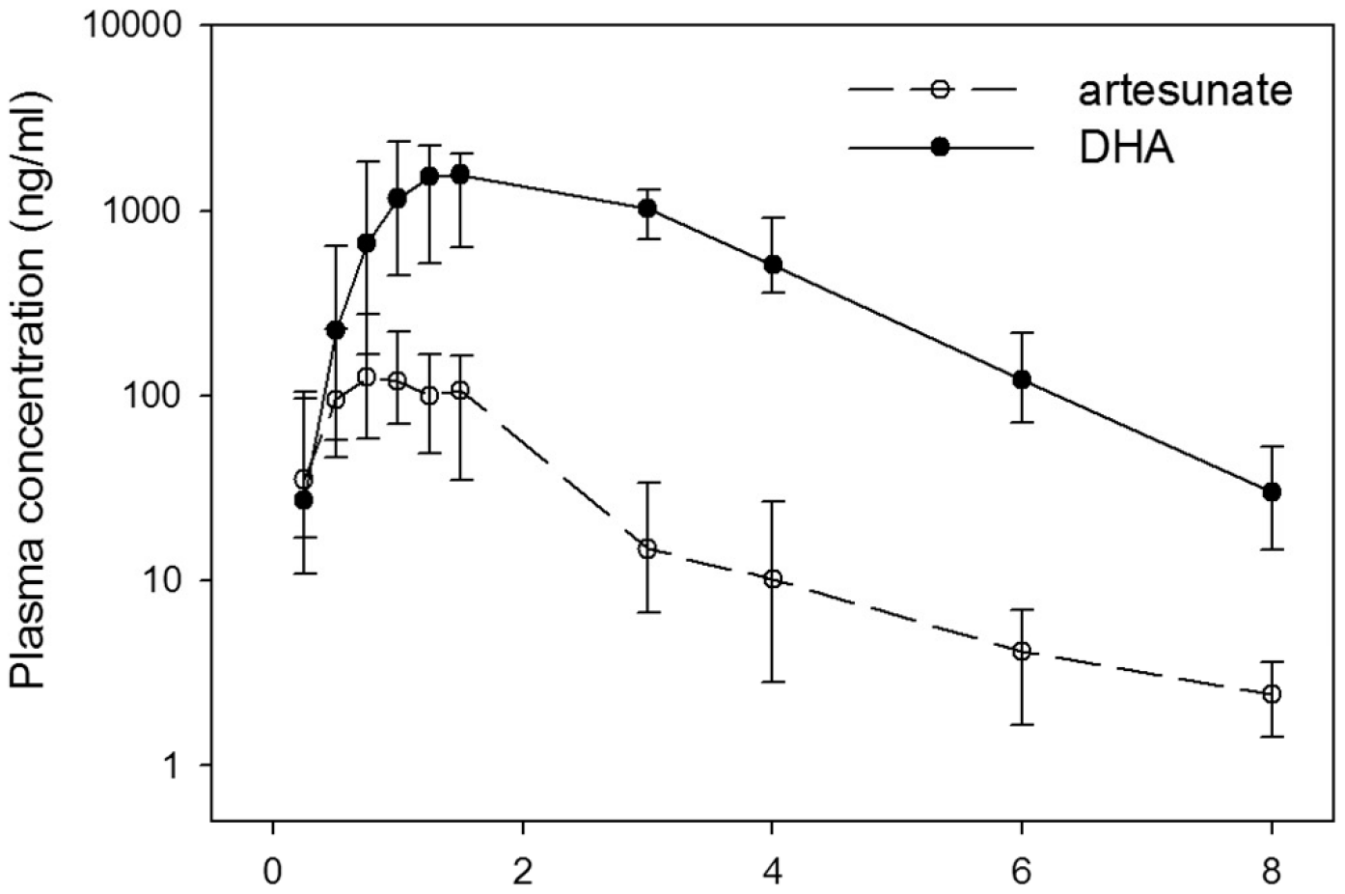
Plasma concentration-time curve of artesunate (dashed line) and DHA (solid line).

The time-plasma concentration-time plots of artesunate and DHA are displayed in Fig. 7. A participant who vomited and was re-treated was excluded from the pharmacokinetic analysis. The weight-adjusted artesunate doses were similar among participants (median 4 mg/kg; range 3.9–4.6 mg/kg). The maximal concentration, AUC, and elimination half-life of DHA were about 7, 15, and ∼2 times that of artesunate, respectively. The maximal concentration of artesunate (median 274 ng/mL, interquartile range (IQR) 35.5–127 ng/mL) was obtained in less than an hour and the drug disappeared from the plasma with an elimination half-life of 0.5 hours (IQR 0.10–2.3 hours) and total clearance of 13.5 L/hr/kg (IQR 6.84–37.1). DHA reached its maximal concentration (median 1850 ng/ml, IQR 546–4100 ng/mL), and had an elimination half-life of 0.91 hours (IQR 0.46–2.49 hours) and total clearance 0.63 L/hr/kg (IQR 0.34–2.24 L/hr/kg). Artesunate and DHA pharmacokinetics were not significantly different between those with (Fig. S5A) and without (Fig. S5B) persistent parasitemia at 72 hours, or between fast and slow clearers (data not shown).

## Discussion

This study provides evidence of decreased sensitivity of *P. falciparum* to artemisinins in Myanmar, in a subset of the parasite population in southern Myanmar near the Thailand border. Although this is the first report of artemisinin resistance originating within Myanmar, a progressive decline in parasite response, over-time from 2001 to 2010, to various artesunate-based regimens has been reported in patients with hyperparasitemia in Thailand along the northwestern border of Thailand with Myanmar [16]. Varying patterns of parasite clearance following oral artesunate monotherapy have been reported from different countries in the region. In Bangladesh [24] and Laos [11] where recent studies found no evidence of resistance, most infections cleared in less than 36 hours, and there were no cases of persistent parasitemia after 48 hours. In contrast, in western Cambodia, where artesunate resistance has been confirmed, more than 40% of cases had dramatically delayed parasite clearance [2]. Although the distribution pattern of parasite clearance time in our study population (Fig. 6) showed that the majority of infections cleared as quickly as those in Bangladesh and Laos, more than 1/3 of falciparum infections were “slow clearers” based on a recently published threshold of parasite clearance half-life of 6.2 hours [16], and 10% of falciparum cases took 84–96 hours to clear. This slow-clearing subpopulation of southern Myanmar parasites cleared more slowly than the slowest-clearing infections in Wang Pha, Thailand, near the northwestern Myanmar-Thailand border [16], similar to the slowest-clearing infections in western Cambodia [2], [3], where this new form of artemisinin-resistant malaria is thought to have first emerged [2], [16], [25], [26]. Slow parasite clearance seen in this study is thought to represent the first manifestation of artemisinin resistance. In western Cambodia significant decreases in ACT treatment efficacy of both uncomplicated and severe malaria are now being seen [13]. These findings in Myanmar are concerning in the context of progressively decreasing ACT efficacy and increasing parasite clearance time observed over time on Myanmar’s eastern border with Thailand [15], [16], and are consistent with either the spread of artemisinin-resistant falciparum malaria from western Cambodia or the independent emergence of such parasites in Myanmar. Studies are now underway at additional sites in Myanmar to assess the extent and distribution of this phenomenon. Gene flow studies to map parasite migration patterns are also planned to determine whether the slow-clearing parasites spread to Myanmar from Cambodia or whether they arose *de novo* in Myanmar.

We also observed a high rate of persistent parasitemia 72 hours after artesunate monotherapy, despite the 100% PCR-corrected 28-day clinical efficacy. While the 100% cure rate we observed indicates that artemisinins retain good efficacy and ACTs can still be used in Myanmar at present, this apparently high efficacy of artemisinin in our study should be interpreted with caution. In our study, the participants were followed only 28 days, instead of 42 days, and we may have missed cases of late treatment failure. In addition, the study was not adequately powered to detect the true efficacy of artesunate. Of note, we do not endorse the use of artesunate monotherapy for any purpose other than in clinical trials designed to assess the independent ability of artemisinins, to clear P. falciparum without confounding by the effect of a partner drug.

One limitation of our study was that the frequency of blood smear examination was every 12 hours, while most other recent studies (except the Bangladesh study) sampled parasitemia every 6–8 hours. This relatively infrequent observation carries apparent potential for over-estimation of the time required to clear parasites in some cases and misinterpretation of the observed bimodal distribution of the rate of clearance. Nevertheless, our data can be directly compared with the Bangladesh data, and our findings are in striking contrast to those from Bangladesh, where almost all infections cleared in fewer than 36 hours [24]. In contrast, more than 50% of our study participants remained parasitemic at 48 hours, and 27% still had microscopically detectable parasites at 72 hours, providing strong evidence of abnormally slow parasite clearance at our study site. Drug quality, compliance with study drug intake, and inadequate pharmacokinetics of artesunate or DHA, are all unlikely to have contributed significantly to the observed variability in parasite clearance, since all artesunate doses were administered under direct observation, and the pharmacokinetics of artesunate and DHA were comparable to previous observations in the region [27]–[29], and not associated with delayed parasite clearance. We did not evaluate the contribution of other host factors in the variability of parasite clearance, such as immunity or hemoglobinopathies, which might affect antimalarial treatment efficacy.

Our observation of highly frequent *P. vivax* infections is consistent with other studies reporting that *P. vivax* was a common cause of recurrent parasitemia following treatment of acute falciparum malaria in this region [30]–[32]. The higher rate of recurrent vivax malaria in our study than in others may be explained by the use of short-acting artesunate alone with no partner drug [30] and the presence of sub-microscopic vivax parasitemia at enrollment that was likely suppressed by high-density falciparum parasitemia. Following artesunate treatment and clearance of *P. falciparum*, relapse of vivax infection (release of liver-stage hypnozoites of *P. vivax*, which are unaffected by artesunate treatment of blood-stage parasites) may have occurred after the falciparum infection subsided and artesunate was eliminated from the body. With growing recognition of the public health significance of *P. vivax*, arguments have been made for presumptive radical treatment of *P. vivax* using primaquine after all *P. falciparum* treatments in settings where mixed infections are common [31]. Additional research is needed to guide recommendations on the use of primaquine for radical elimination of *P. vivax* and for its antigametocyticidal effect on *P. falciparum.*

This study provides credible evidence of reduced susceptibility of *P. falciparum* parasites to artemisinins in Myanmar, adjacent to its southern border with Thailand. Myanmar has a population of approximately 58 million people with diverse ethnic backgrounds, more than half of whom occupy remote rural areas where malaria is highly endemic, including areas bordering Thailand, Laos, Bangladesh, India, and China. Although Myanmar has only 4% of Southeast Asia’s population, it has 20% of the region’s malaria burden [33]. Recognizing the high mobility of border populations and the risk of spread of possible resistance, the Myanmar Artemisinin Resistance Containment (MARC) [34] project was initiated in 2011 by the WHO and Myanmar partners in areas of suspected artemisinin resistance, aiming to minimize its possible spread by implementing early and effective diagnostic, therapeutic and preventive measures. Studies are also ongoing to elucidate the genetic mechanism(s) of artemisinin resistance [35] in hopes of identifying molecular markers that can be used as tools for surveillance. The threat of artemisinin resistance so far appears to be confined to a relatively small part of Southeast Asia, and artemisinin efficacy remains unaltered in most other parts of the world [10]. The loss of artemisinins to drug resistance would be disastrous for global malaria control, as occurred with the emergence and spread of chloroquine resistance [36], [37]. More comprehensive mapping of the present extent and patterns of spread of resistance are urgently required for the optimization of regional containment efforts.

## Supporting Information

Figure S1 **Map of Myanmar.** The red star indicates the location of study site, Kawthaung.
(TIF)

Figure S2 **Lack of association between parasite clearance rate and initial parasite density.**
(TIFF)

Figure S3 **Lack of association between parasite clearance rate and maximal concentration of dihydroartemisinin.**
(TIFF)

Figure S4 **Lack of association between parasite clearance rate and total exposure of dihydroartemisinin.**
(TIFF)

Figure S5 **Plasma concentration-time curve of artesunate (Panel A) and DHA (Panel B) in participants with (dashed line) and without (solid line) persistent parasitemia 72 hours after artesunate treatment.**
(TIFF)

Protocol S1 **Trial protocol.**
(DOC)

Checklist S1 **CONSORT checklist.**
(DOC)
