# Supplementary figures and images for: Reduced Susceptibility of Plasmodium falciparum to Artesunate in Southern Myanmar
Source: PLoS One. 2013 Mar 8;8(3):e57689. doi: 10.1371/journal.pone.0057689 (PMC3592920; doi:10.1371/journal.pone.0057689)

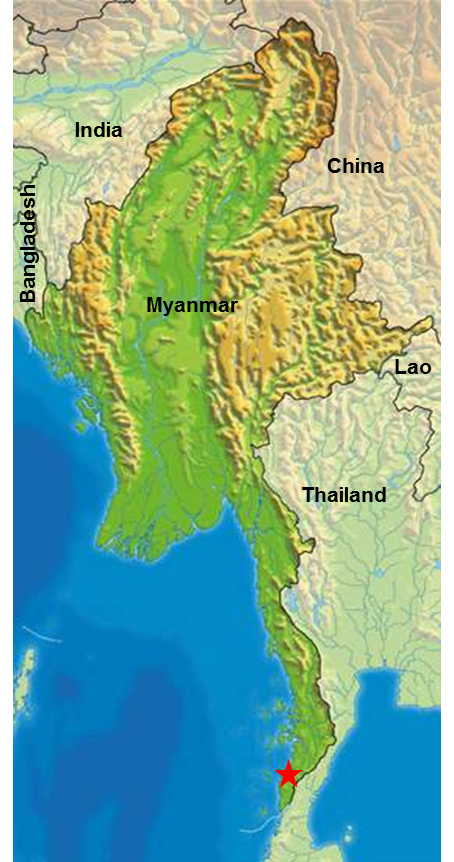

Supplement: Figure S1 — Map of Myanmar. The red star indicates the location of study site, Kawthaung. (TIF) [file pone.0057689.s001.tif]

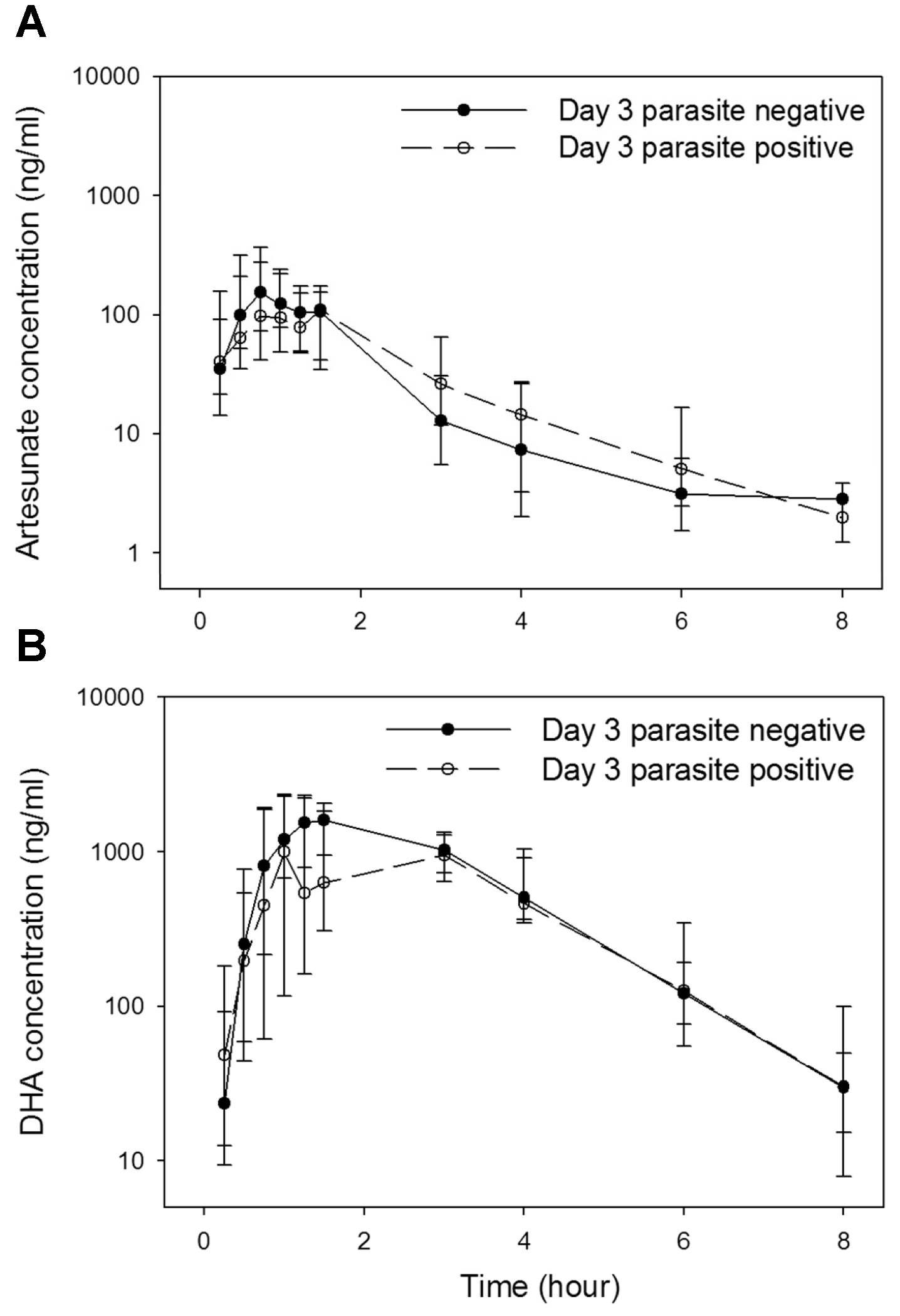

Supplement: Figure S5 — Plasma concentration-time curve of artesunate (Panel A) and DHA (Panel B) in participants with (dashed line) and without (solid line) persistent parasitemia 72 hours after artesunate treatment. (TIFF) [file pone.0057689.s005.tiff]
