# Supplementary material for: Reduced Susceptibility of Plasmodium falciparum to Artesunate in Southern Myanmar
Source: PLoS One. 2013 Mar 8;8(3):e57689. doi: 10.1371/journal.pone.0057689 (PMC3592920; doi:10.1371/journal.pone.0057689)
Supplement: Protocol S1 — Trial protocol. (DOC) [file pone.0057689.s006.doc]

| Cover sheet for therapeutic efficacy test protocol | |
| --- | --- |
| Title | Efficacy artesunate 7days therapy for the treatment of uncomplicated *Plasmodium falciparum* malaria in Kawthaung (Tanintharyi Division) in Myanmar. |
| Protocol submission date | 1 October 2010 |
| Protocol number | Myanmar 4/2010 v2 |
| Principal investigator | Dr. Myat-Phone-Kyaw  Deputy Director/Head, Parasitology research division  Department of Medical Research (Lower Myanmar)  Tel: 951-3754 47, 951375449, 951-375457  Fax: 951 251514  Email: [myat@mptmail.net.mm](mailto:myat@mptmail.net.mm) |
| Co-investigator | Dr. Ye Htut  Deputy Director General  Department of Medical Research (Lower Myanmar) |
| Co-investigator | Dr. Khin Mon Mon  Deputy Director, Vector Borne Disease Control (VBDC)  Department of Health (DOH |
| Co-investigator | Dr. Thaw Zin (Pharmacology)  Department of Medical Research (Lower Myanmar) |
| Co-investigator | Dr. Min Wun (Pharmacology)  Department of Medical Research (Lower My anmar) |
| Co-investigator | Dr. Kay Thwe Han (Molecular Biology)  Department of Medical Research (Lower Myanmar) |
| Medical monitor | Dr. Leonard Ortega  WHO representative (Acting) Medical Advisor,  Malaria and Other Mosquitoes Borne Diseases  WHO Country Office for Myanmar  Telephone: 951 212606 / 8/ 9, 226895  Fax: 951 2120605, 202365,E-mail: [ortegal@searo.who.int](mailto:ortegal@searo.who.int) |
| Participating institutions | Department of Health  Tel: 95 1 375447  Fax: 95 1 251514 |
| Participating institutions | Mahidol Oxford Research Unit, Bangkok |
| Study dates | From October 2010 to September 2011 |
| Sponsor | Department of Medical Research (Lower Myanmar) No.5, Ziwaka Road, Dagon P.O. Yangon 11191, Myanmar |

Summary

Title: Efficacy of artesunate 4 mg/day over 7 days for the treatment of uncomplicated *Plasmodium falciparum* malaria in Kawthaung (Tanintharyi Division) in Myanmar.

Background: The artemisinin resistance is not just on the Thai-Cambodia border, but may have extended to or emerged on the Thai-Myanmar, China-Myanmar and Cambodia-Viet Nam borders, confirmatory artemisinin monotherapy in vivo studies and molecular fingerprinting of parasite genomes to track artemisinin resistance (gene flow) moving around the region is even more important now. Of the data presented from these countries, the slow parasite clearance time (PCT) following treatment with artemisinin derivatives was of greatest concern. With combination therapy, artesunate usually provides the initial rapid decrease of PCT, with 95% of patients clearing peripheral parasitaemia within 48 hours, hence if the PCT is slow despite the drug being in the blood at adequate levels, this could provide evidence that it is failing.

Objective: To assess the efficacy artesunate 4 mg/day over 7 days for the treatment of uncomplicated *Plasmodium falciparum* malaria in Kawthaung (Tanintharyi Division) in Myanmar.

Methods: An antimalarial drug efficacy trial will be conducted in Kawthaung (Tanintharyi Division), Mzanmar. The participants will be febrile people > 18 years with confirmed uncomplicated *P. falciparum* infection. Patients will be treated with artesunate 28 mg/kg over 7 days. Clinical and parasitological parameters will be monitored over a 28-day follow-up period to evaluate drug efficacy. The study will be conducted from October 2010 to September 2010. The results of this study will be used to assist the Ministry of Health of Myanmar in assessing the current national treatment guidelines for uncomplicated *P. falciparum* malaria and mapping the extent of artesunate resistance.

1. Background

The current national treatment guideline for the treatment of uncomplicated malaria is artemether-lumefantrne, dihydroartemisinin-piperaquine and artesunate-mefloquine. An antimalarial drug efficacy trial was conducted in 4 sentinel sites (Kayin, Mon, Kachin and Yakhine States) in Myanmar between June and November, 2007. Patients were treated with artesunate-amodiaquine combination and artemether-lumefantrine combination. A total of 535 subjects (171 subjects in Yakhine, 71 subjects in Kayin, 151 subjects in Kachin, and 142 subjects in Mon state) were enrolled for therapeutic efficacy study. 296 subjects were treated with artemether-lumefantrine and 239 subjects with artesunate-amodiaquine after random selection. In in artesunate-amodiaquine group there was one late parasite failure case each in Kachin, Kayin and Mon State. In artemether-lumefantrine group there was one late parasite failure (LPF) at day 14 in Kayin State, 2 late treatment failure cases each in Mon state and Yakhine State. The late treatment failure cases were compared with genotypic profiles of the pre- and post parasite strains by the malaria parasite genes, msp1, msp2 and glurp. After molecular analysis, one patient from Kachin was re-infected case and the other two (Kayin & Mon) were true recrudescence in artesunate-amodiaquine group, and those from Yakine are re-infected cases and 1 from Kayin, 2 from Mon State were true recrudescence to artemether-lumefantrine combination. In a 2008 clinical trial of 3-day artemisinin-piperaquine (Artequick™) at the Defense Services General Hospital, delayed parasite clearance time of >100 hours in 7/50 patients and >90 hours in 2 patients was also observed.

Recently, longer parasite clearance and decreasing efficacy of ACTs were observed in Shwe Kyin and Kawthaung, on the south eastern tip of Myanmar bordering with Ranong, Thailand. A total of 168 cases were completed for study in artemether-lumefantrine group and 153 in dihydroartemisinin-piperaquine group. There was one ETF case at Shwe Kyin, who had persistence of parasitemia up to day 3 with parasite count more than 10% of the day 0 count. Clinically 8 recurrence cases in artemether-lumefantrine group and 4 cases in dihydroartemisinin-piperaquine group were noted, but reinfection can be excluded after molecular analysis. The persistant of parasitaemia was noted in all study sites for both combinations and marked elevation was noted in patients treated with dihydroartemisinin-piperaquine at Kawthaung (18.5%). This same site in Thailand has been showing increasing artesunate-mefloquine failures since 2006. The presented results of that therapeutic efficacy trials in Cambodia and Thailand were the failure rates of combinations with artemisinin derivatives and mefloquine and artemether-lumefantrine in both countries, as well as increased parasite clearance time (PCT) from clinical trials using artesuante-mefloquine and artesunate+pyronaridine in Cambodia. Of the data presented from the two countries, the slow PCT following treatment with artemisinin derivatives was of greatest concern. With combination therapy, artesunate usually provides the initial rapid decrease of PCT, with 95% of patients clearing peripheral parasitaemia within 48 hours, hence if the PCT is slow despite the drug being in the blood at adequate levels, this could provide evidence that it is failing. The resistance monitoring shows the proportion of patients who are parasitaemic on day 3 is the best measure of slow parasite clearance from available clinical trial data and should be used as an early warning system for development of artemisinin resistance.

The critical question is whether artemisinin derivatives are actually failing or not against current recommended total dosage (12 mg/kg) or higher dosage. Since artemisinin resistance is not just limited to the Thai-Cambodia border, but may have extended to or emerged on the Thai-Myanmar, China-Myanmar and Cambodia-Viet Nam borders, confirmatory artemisinin resistance through monotherapy in vivo studies and molecular fingerprinting of parasite genomes to track artemisinin resistance (gene flow) moving around the region is even more important now.

The trial described here proposes to assess the PCT and the efficacy after 4mg/kg oral artesunate over 7 days. The results of this study will be compared to recent data from patients in Cambodia and Thailand treated with equivalent doses of artemisinin derivatives.

2. Objectives

The primary objective of this study is to assess the PCT and efficacy of artesunate 4mg/kg/day in the treatment of uncomplicated *P. falciparum* malaria in Kawthaung (Tanintharyi Division) in Myanmar.

The specific objectives are:

- to measure the PCT with blood sampling for parasite counts twelve hourly and to measure clinical and parasitological efficacy of artesunate 4mg/kg/day over 7 days in patients aged > 18 years, suffering from uncomplicated falciparum malaria
- to determine the proportion with early treatment failures, late clinical failures, late parasitological failures or adequate clinical and parasitological response as indicators of efficacy
- to differentiate recrudescence from new infection by polymerase chain reaction (PCR) analysis
- to evaluate the number of adverse events
- to formulate recommendations and to enable the Ministry of Health to make informed decisions about whether the current national antimalarial treatment guidelines should be updated. The results of this study will indicate if artemisinin resistance is present in Myanmar which allows a better mapping of the situation of artemisinin resistance in the region and indicate if containment effort should be started.

The secondary objectives are:

- to determine the polymorphism of molecular markers for antimalarial resistance; and
- to determine the blood concentration of artesunate and dihydroartemisinin

3. Methods

3.1 Study design

People with uncomplicated malaria presenting at the Kamaukkyi 38 mile station hospital who meet the study inclusion criteria will be enrolled and treated on site with artesunate 4mg/kg/day until PCT and monitored for 28 days. The follow-up will consist of a fixed schedule of check-up visits and corresponding clinical and laboratory examinations. On the basis of the results of these assessments, the patients will be classified as having therapeutic failure (early or late) or an adequate response. The proportion of patients experiencing therapeutic failure during the follow-up period will be used to estimate the efficacy of the study drug(s). PCR analysis will be used to distinguish between a true recrudescence due to treatment failure and episodes of reinfection.

3.2 Study site

Tanintharyi Division lies at the southern end of Myanmar. A narrow strip of coast between Thailand on the east and the Andaman Sea on the west, Tanintharyi extends south for c.400 mi (650 km) from Mon State to the Isthmus of Kra and includes many offshore. The area of the Division is 16,735 square miles. Out of about 1,000 islands along Myanmar's coastline over 800 are in Tanintharyi coast. Myeik is the Capital of Tanintharyi Division with three districts -- Dawei, Myeik and Kawthoung -- formed with ten Townships and 317 Wards and 328Village Tracts and the population is about 917,628. Evergreen forests thrive in the region as it is located in the tropical climate zone with torrential rains. As the land is mountainous, it is a thinly populated area. Majority of the residents of the Division live along the coastal plains and river valleys. Kawthaung District has high malaria mortality and morbidity, also a high risk area that was selected to study. Kamaukkyi Station hospital, Kawthaung, in Tanintharyi Division has been selected for the study where the falciparum malaria patients will be hospitalized for 7 days and follow-up activities will be done.

3.3 Study population

The population will consist of patients with uncomplicated *P. falciparum* malaria attending the study health clinic who are aged > 18 years. All adult patients will sign an informed consent form for participation. Patients will be hospitalized for the 7 first days.

3.4 Timing and duration of study

The study will be conducted during the malaria transmission season, from October 2010 to September 2011.

3.5 Inclusion criteria

- age > 18 years;
- mono-infection with *P. falciparum* detected by microscopy;
- parasitaemia of 10 000-100 000/µl asexual forms;
- presence of axillary ≥ 37.5 °C or oral or history of fever during the past 24 h;
- ability to swallow oral medication;
- ability and willingness to comply with the study protocol for the duration of the study and to comply with the study visit schedule; and

3.6 Exclusion criteria

- presence of signs of severe falciparum malaria according to the definitions of WHO (Appendix 1);
- mixed or mono-infection with another Plasmodium species detected by microscopy;
- presence of severe malnutrition (defined as a child whose growth standard is below –3 z-score, has symmetrical oedema involving at least the feet or has a mid-upper arm circumference < 110 mm);
- presence of febrile conditions due to diseases other than malaria (e.g. acute lower respiratory tract infection, severe diarrhoea with dehydration) or other known underlying chronic or severe diseases (e.g. cardiac, renal and hepatic diseases, HIV/AIDS);
- regular medication, which may interfere with antimalarial pharmacokinetics;
- history of hypersensitivity reactions or contraindications to any of the medicine(s) being tested or used as alternative treatment(s); and
- a positive pregnancy test or breastfeeding (include this criterion only if adults are included);
- unable to or unwilling to take a pregnancy test or contraceptives (for women of child-bearing age);
- previous antimalarial drug intake in the past 48 hours;
- patients presenting with spenectomy.

3.7 Loss to follow-up

Loss to follow-up occurs when, despite all reasonable efforts, an enrolled patient does not attend the scheduled visits and cannot be found. No treatment outcome will be assigned to these patients. Every effort must be made to schedule a follow-up visit for patients who fail to return to the study site, especially during but also after administration of the study drug. These patients will be classified as lost to follow-up and censored or excluded from the analysis. Patients who are lost to follow-up but who subsequently return to the study site before day 28 will not be turned away and will be encouraged to return for check-up visits. The principal investigator will decide whether the patient is to be definitely classified as lost to follow-up on the basis of his or her history or is to be maintained for the analysis.

3.8 Patient discontinuation or protocol violation

Study patients who meet any of the following criteria will be classified as withdrawn.

- withdrawal of consent. A patient may withdraw consent at any time, without prejudice for further follow-up or treatment at the study site.
- failure to complete treatment, due to:
- persistent vomiting of the treatment. A patient who vomits the study medication twice will be withdrawn from the study and given rescue treatment.
- failure to attend the scheduled visits during the first 3 days; or
- serious adverse events necessitating termination of treatment before the full course is completed. A patient can be discontinued from the study if the principal investigator decides so due to an adverse event of adequate nature or intensity. In this case, information on the adverse event and symptomatic treatment given must be recorded on a case report form. If the adverse event is serious, the principal investigator must notify the sponsor or its designee immediately and follow the reporting procedures described in section 5.3.
- enrolment violation:
- severe malaria on day 0; or
- erroneous inclusion of a patient who does not meet the inclusion criteria.
- voluntary protocol violation: self- or third-party administration of antimalarial drug (or antibiotics with antimalarial activity) (Appendix 2);
- involuntary protocol violation:
- occurrence during follow-up of concomitant disease that would interfere with a clear classification of the treatment outcome;
- detection of mono-infection with another malaria species during follow-up; or
- misclassification of a patient due to a laboratory error (parasitaemia), leading to administration of rescue treatment.

Patients who are withdrawn will nevertheless be followed up until recovery or the end of follow-up, if possible; however, no treatment outcome will be assigned to these patients, and they will be censored or excluded from the analysis. The reasons for discontinuation or protocol violation will be recorded on the case report form.

4. Treatment

4.1 Antimalarial treatment

The patients will be received artesunate 50 mg per tablet (Guilin Pharmaceutical Company, PRC) 4mg/kg/day for 7 days. Each dose administration will be observed and recorded. The correct drug dosage will be determined from the dosing chart (Appendix 3).

All doses of medicine will be administered under the supervision of a qualified member of the staff designated by the principal investigator. The study patients will be observed for 30 min after medicine administration for adverse reactions or vomiting. Any patient who vomits during this observation period will be re-treated with the same dose of medicine and observed for an additional 30 min. If the patient vomits again, he or she will be withdrawn and offered rescue therapy.

4.2 Concomitant treatment and medication that should not be used

Fever over 38 °C can be treated with paracetamol or acetaminophen.

During follow-up, if infections other than malaria require the administration of medicines with antimalarial activity, the patient will be withdrawn from the study. Patients given tetracycline as an eye ointment will not be excluded (Appendix 2). Patients will be withdrawn from the study in the case of self-medication or if an antimalarial drug or an antibiotic with antimalarial activity is administered by a third party.

Adverse events requiring treatment can be treated according to local practice. If there is a clinical indication for any additional medication during the course of the study, including medication given to treat an adverse event related to the study medicine, the name of the medicine, the dosage and the date and time of administration must be recorded on the case report form.

The use of herbal remedies during the study should be avoided, and participants should be encouraged to return to the study site for treatment if they feel unwell. If any herbal remedies are taken during the study, this should be captured on the case report form, under ‘study medication administration’.

4.3 Rescue treatment

If a patient meets one of the criteria for therapeutic failure, ETF and LTF cases, he/she will receive the other artemether-lumefantrine. If the patient is reinfected with another malaria species (*P. vivax, P. malariae or P. ovale*) he/she will receive chloroquine total dose of 25mg base/kg over three days, followed by primaquine 0.25mg/kg/day for 14 days (except for *P. malariae*) according to the current national recommendations.

If the patient vomits the treatment twice, he/she will receive parenteral therapy with artemether IM (80mg/ amp) injection 160mg at 0hrs and l2th hours, and 80 mg at 24th and 48th hours (total = 480mg) plus oral mefloquine (500mg) x 2 doses at 48th and 54th hours and be withdrawn from the study.

With any sign of severe malaria, the study patient will be treated with quinine injection 8 mg/kg of quinine base every 8 hours. Quinine will be given 3 time a day until the patient can reliably take oral medication. The patient will then continue on oral quinine (3x8 mg/kg/day) until they have completed seven days treatment. A seven days course of doxycycline (3 mg/kg/day) will be given also to patients as well as relevant supportive treatments.

5. Evaluation criteria

The study end-point is the classification assigned to a patient. Valid study end-points include: the number of patients parasite positive at day 3, PCT, parasite reduction rate (slope of the log-linear parasite clearance curve), the parasite reduction rate at 48 hours, treatment failure, completion of the follow-up period without treatment failure, loss to follow-up, withdrawal from study, and protocol violation. At all times, the well-being of the patient will take priority over his or her continuation in the study. Secondary end-point is the pharmacokinetic profile of the patients.

5.1 Efficacy and safety evaluation

### 5.1.1 Classification of treatment outcomes

Artesunate should produce parasite reduction ratios of approximately 10,000/cycle, which result in parasite clearance times, which are usually less than 48 hours. This is a descriptive study; its purpose will be a detailed analysis of how the group responds.

Treatment outcomes will be classified on the basis of an assessment of the parasitological and clinical outcome of antimalarial treatment according to the latest WHO guidelines.[[1]](#footnote-2) Thus, all patients will be classified as having early treatment failure, late clinical failure, late parasitological failure or an adequate clinical and parasitological response, as defined in Appendix 4.

As parasitological cure is the goal of antimalarial therapy, all study patients who show treatment failure will be given rescue treatment. Follow-up will continue until recovery. The results from these patients do not need to be recorded systematically for the purpose of the surveillance study.

### 5.1.2 Safety end-points

The number of any adverse event will be documented. All patients will be asked routinely about previous symptoms and about symptoms that have emerged since the previous follow-up visit. When clinically indicated, patients will be evaluated and treated appropriately. All adverse events will be recorded on the case report form. Serious adverse events (see definitions in 5.3) must be reported to the sponsor with 48 hours.

5.2 Clinical evaluation

All patients will be evaluated clinically as described below.

### 5.2.1 Physical examination

A standard physical examination will be performed at baseline (day 0 before dosing) and on days 1, 2, 3, 7, 14, 21, and 28. A complete medical history, demographic information and contact details will be taken at baseline.

### 5.2.2 Body weight

Body weight will be recorded on day 0 to the nearest kilogram on a Salter scale. The scales will be properly calibrated. Patients should not wear excessive clothing while being weighed as this can overestimate their true weight. The screening weight will be used to satisfy the inclusion or exclusion for nutrition status as well as to calculate the dose (number of tablets) to be administered. The reliability of the scales will be verified before the study begins and checked at regular intervals.

### 5.2.3 Body temperature

Axillary, oral temperature will be measured at baseline (day 0 before dosing) and on days 1, 2, 3, 7, 14, 21, and 28. Temperature will be measured with a thermometer that has a precision of 0.1 °C. Temperature will also be measured as clinically indicated. If the result is < 36.0 °C, the measurement will be repeated. The same route should be used throughout the study.

The quality of the temperature-taking technique and the thermometers should be assessed regularly. Thermometers should be tested in a water-bath of known temperature before the study begins and at regular intervals thereafter.

### 5.2.4 Microscopic blood examination

Thick and thin blood films for parasite counts will be obtained and examined at screening on day 0 to confirm adherence to the inclusion and exclusion criteria. Thick or thin blood films will be also examined every 12 hours until PCT and on days 1, 2, 3, 4, until negativation, 7 (if after PCT), 14, 21, 28 or on any other day if the patient returns spontaneously and parasitological reassessment is required. Specimens will be labelled anonymously (screening number or study number, day of follow-up, date).

A fresh Giemsa stain dilution will be prepared at least once a day and possibly more often, depending on the number of slides to be processed. Giemsa-stained thick and thin blood films will be examined at a magnification of 1000× to identify the parasite species and to determine the parasite density.

Three blood slides per patient will be obtained: two thick blood smears and one thin blood smear. One slide will then be stained rapidly (10% Giemsa for 10–15 min) for initial screening, while the others will be retained. If the patient is subsequently enrolled, the second slide will be stained more carefully (e.g. 2.5–3% Giemsa for 45–60 min), and slower staining will also be used for all slides obtained at follow-up visits. The study number of the patient, the date and the day of follow-up will be recorded either on the frosted edge of the slide or on the glass with a permanent glass pen.

The thick blood smear for initial screening will be used to count the numbers of asexual parasites and white blood cells in a limited number of microscopic fields. The adequate parasitaemia for enrolment is at least one parasite for every six white blood cells, corresponding to approximately 1000 asexual parasites per microlitre.

The second blood smear will be used to calculate the parasite density, by counting the number of asexual parasites in a set number of white blood cells (typically 200) with a hand tally counter. Once a field has been started, it must be counted to completion; the final number of white blood cells will therefore rarely be exactly 200. If more than 500 parasites have been counted before 200 white blood cells have been reached, the count will be stopped after the reading of the last field has been completed. Parasite density, expressed as the number of asexual parasites per µl of blood, will be calculated by dividing the number of asexual parasites by the number of white blood cells counted and then multiplying by an assumed white blood cell density (typically 6000 per µl).

|  | Parasite density (per µl) = number of parasites counted × (6000) |  |
| --- | --- | --- |
|  | Number of leukocytes counted |  |

The same technique will be used to establish the parasite count on each subsequent blood film. When the number of asexual parasites is less than 10 per 200 white blood cells in follow-up smears, counting will be done against at least 500 white blood cells (i.e. to completion of the field in which the 500th white blood cell is counted). A blood slide will be considered negative when examination of 1000 white blood cells reveals no asexual parasites. The presence of gametocytes on an enrolment or follow-up slide will be noted, but this information will not contribute to basic evaluation.

In addition, 100 fields of the second thick film will be examined to exclude mixed infections; in case of any doubt, the thin film will be examined for confirmation. If examination of the thin film is not conclusive, the patient will be excluded from the analysis after complete treatment and follow-up.

Two qualified microscopists will read all the slides independently, and parasite densities will be calculated by averaging the two counts. Blood smears with discordant results (differences between the two microscopists in species diagnosis, in parasite density of > 50% or in the presence of parasites) will be re-examined by a third, independent microscopist, and parasite density will be calculated by averaging the two closest counts.

### 5.2.5 Genotyping of falciparum parasites

In order to differentiate a recrudescence (same parasite strain) from a newly acquired infection (different parasite strain), a genotype analysis will be conducted. This is based on the extensive genetic diversity among the malaria parasite genes *msp1*, *msp2* and *glurp*.[[2]](#footnote-3) The genotypic profiles of pre- and post-parasite strains are compared.

In order to minimize discomfort to the patient due to repeated finger pricks, two to three drops of blood will be collected on filter paper Whatman 3MM during enrolment and each time blood smears are required according to the protocol on and after day 7.

Specimens will be labelled anonymously (study number, day of follow-up, date), kept in individual plastic bags with desiccant pouches and protected from light, humidity and extreme temperature until analysed. When these conditions cannot be achieved, for example in extremely humid environments where air-conditioning is not available, storage in a refrigerator or freezer may be considered, but great care must be taken to protect samples from frost and moisture. The techniques used are polymerase chain reaction, PCR-RFLP and sequencing. The tests will be done at the laboratory of Department of Medical Research, lower Myanmar. Paired filter papers will be used for parasite DNA extraction and genotyping only in cases of treatment failure. Unused filter papers will be destroyed immediately after the study.

### 5.2.6 Molecular markers for antimalarial drug resistance

*P. falciparum* isolates will be collected for whole-genome sequencing. The samples will be genotyped for close to one million SNPs (single nucleotide polymorphisms) and will contribute to creating an overall picture of fine-scale global genetic variation of *P. falciparum*. This project is designed to uncover unique genetic signatures present in the Myanmar *P. falciparum* parasite population.

At admission to the trial, a single blood sample of 5 ml will be collected into EDTA tubes. Host white blood cells will be removed using a Lymphoprep (Axis-Shield) separation and PlasmodiPur filtration (Eurodiagnostika, The Netherlands) step. The parasite DNA will be extracted using DNA Blood Maxi Kits (QIAGEN). Blood samples will be transported to the Wellcome Trust to the Mahidol Oxford Research Unit in Bangkok and will be shipped to the Wellcome Trust Sanger Institute, UK at the end of the clinical trial.

### 5.2.7 Antimalarial drug blood concentration

A total of 1 ml for determining the blood concentration of artesuante and dihydroartemisinin will be collected at according to the timetable below from an indwelling catheter. Specimens will be labelled anonymously (study number, day of follow-up, date). One ml of whole blood will be collected in appropriate collection tubes containing sodium fluoride/potassium oxalate as anticoagulant. The tubes will be pre-chilled on wet ice prior to use. After collection of blood, the tube should be placed on wet ice and processed as soon as possible after collection (the samples can be placed in the ice bath for 5 to 10 minutes prior to centrifugation to allow the blood to chill in the tube). Centrifuge blood within 15 minutes of collection. Centrifuge whole blood at (4°C) 2000 x g for 7 minutes to obtain plasma. Immediately after centrifugation, transfer plasma into a screw cap cryovial (Nalgene No.: 5000 0012 or suitable alternative) and freeze the plasma samples at or below - 80°C in a laboratory freezer until analysis at the ned of the clinical trial.The quantification of artesunate and dihydroartemisinin using LC-MS/MS and the pharmacokinetic analysis will be conducted at the clinical pharmacology laboratory of Mahidol Oxford Research Unit in Bangkok in Thailand. Samples will be batched and shipped for analysis upon completion of the study.

| Day | Hour | | | | | | | | | | |
| --- | --- | --- | --- | --- | --- | --- | --- | --- | --- | --- | --- |
| 0 | 0 | 0.25 | 0.5 | 0.75 | 1 | 1.25 | 1.5 | 3 | 4 | 6 | 8 |
| Volume of blood (ml) | 1 | 1 | 1 | 1 | 1 | 1 | 1 | 1 | 1 | 1 | 1 |

### 5.2.8 Pregnancy test

Female patients of child-bearing age, defined as those who menstruate and are aged > 18 years, will be asked to take a urine pregnancy test before enrolment in the study, because artesunate is contraindicated during the first trimester. They will also be asked to take a urine pregnancy test on day 28 or on early withdrawal from the study.

Female participants of child-bearing age, defined as those who menstruate and are aged > 18 years, and who are sexually active should use barrier contraceptive devices for the duration of the study. Condoms will be provided by the investigator or study team at the time informed consent is obtained, with appropriate counselling about the risks of becoming pregnant and exposing the fetus to the study medicines.

5.3 Safety assessment

Safety will be assessed by recording the nature and number of adverse events and serious adverse events. Adverse events will be assessed by direct questioning. An adverse event is defined as any unfavourable, unintended sign, symptom, syndrome or disease that develops or worsens with the use of a medicinal product, regardless of whether it is related to the medicinal product. All adverse events must be recorded on the case report form.

A serious adverse event is defined as any untoward medical occurrence that at any dose:

- results in death, is life threatening;
- requires hospitalization or prolongation of hospitalization;
- results in a persistent or significant disability or incapacity; or
- is a congenital anomaly or birth defect.

‘Life-threatening’ means that the person was at immediate risk for death; it does not refer to a adverse event that might have caused death if it were more severe. ‘Persistent or significant disability or incapacity’ means that a person’s ability to carry out normal life functions is substantially disrupted.

All serious adverse events occurring during the study must be recorded and reported by the principal investigator to the sponsor, regardless of whether the principal investigator considers the events to be related to the investigated medicine.

The investigator will collect information on all people who become pregnant while participating in this study and will record the information on the appropriate form. The person will also be followed to determine the outcome of the pregnancy. Generally, follow-up will be no longer than 6–8 weeks after the estimated delivery date. Any premature termination of pregnancy will be reported. While pregnancy itself is not considered an adverse event or a serious adverse event, any complication of pregnancy or elective termination for medical reasons will be recorded as an adverse event or a serious adverse event. A spontaneous abortion is always considered a serious adverse event and will be reported as such.

6. Study assessment

6.1 Screening and enrolment

All patients who meet the basic enrolment criteria (age, fever or history of fever, symptoms of malaria, absence of signs of severe malaria, absence of severe malnutrition, pregnancy) during screening will be assigned a consecutive number and evaluated in greater depth by clinical staff. The screening record form (Appendix 5) will be used to record the general information and the clinical observations on each patient being screened. If the patient meets the clinical criteria, he or she will be examined for parasitaemia. Once the patient meets all the enrolment criteria, he or she will be asked for consent to participate in the study.

6.2 Follow-up

Patients who meet all the enrolment criteria will be given a personal identification number and will receive treatment only after the study has been fully explained to them and they have willingly provided informed consent. Any person who decides not to participate in the study will be examined, treated and followed-up by the health facility staff according to the standard of care established by the Ministry of Health.

The basic follow-up schedule is summarized in Appendix 6. A case report form (Appendix 7) and a serious adverse event report form (Appendix 8) will be used to record the general information and clinical observations on each patient enrolled into the study. The appointment schedule will be clearly explained, and a follow-up card with a personal identification number will be provided.

The day a patient is enrolled and receives the first dose of medicine is designated ‘day 0’. All antimalarial treatment will be given by a study team member under supervision. Enrolled patients will be observed for at least 30 min after treatment to ensure that they do not vomit the medicine. If vomiting occurs within 30 min of treatment, the full treatment dose will be repeated. Ancillary treatment, such as antipyretics, will be provided if necessary to patients by the study team and documented on the case report form. Patients with persistent vomiting (i.e. necessitating more than a single repeat dose) will be excluded from the study and immediately referred to the health facility staff for appropriate management.

Thereafter, patients are required to undergo regular clinical reassessment. Blood films for parasite counts will be made twice a day (12 hourly) til parasite clearance and then on day 7 and then weekly for the remainder of the follow-up period, i.e. on days 14, 21, and 28. Patients will be advised to return on any day during the follow-up period if symptoms return and not to wait for the next scheduled visit day. Clinical reassessment will be sufficiently thorough to ensure patient safety and will include assessment not only for potential treatment failure but also for potential adverse reactions to the medicine. Additionally, blood films will be obtained whenever parasitological reassessment is requested by the clinical staff.

Because many medicines have to be given over several days, the initial visits are critical not only for assessing efficacy but also for ensuring patient safety; defaulters at this stage will not have received a complete course of treatment and may be at risk for clinical deterioration. All reasonable efforts will be made to find defaulters to ensure complete treatment. Similarly, the ultimate success of the study rests on minimizing loss to follow-up. While patients are encouraged to return on their own for scheduled follow-up visits, it is essential that provisions be made ahead of time for locating patients at home if they do not attend as requested. This requires obtaining detailed directions to the home during enrolment, and study team members familiar with the community will be responsible for home visits and means of transport for the patients. The schedule of treatment and follow-up examinations given in this protocol must be followed to ensure data integrity. After day 7 patients who fail to return on day 14 but are present on day 13 or 15 (likewise days 20/22, days 27/29) may still be included in the analysis. Deviation from the protocol of more than 1 day should, however, be avoided (see also section 3.7).

7. Data management

The principal investigator will ensure that the study protocol is strictly adhered to and that all data are collected and recorded correctly on the case report form. Laboratory and clinical data will be recorded on a daily basis on the case report form designed for the study. Data derived from source documents should be consistent with the source documents, or the discrepancies should be explained. Any change or correction to a case report form should be dated and explained and should not obscure the original entry. All case report forms will be checked for completeness.

After the study has been completed, data will be entered into a database by double independent data entry, according to WHO standard procedures.[[3]](#footnote-4) The trial data will be stored in a computer database, maintaining confidentiality.

The principal investigator is responsible for keeping all screening forms, the case report form and the completed subject identification code list in a secure location.

8. Statistical methods

8.1 Sample size

The estimated prevalence of patients positive at day 3 after artesunate 4 mg/kg/day over 7 day in the area is 15%. At a confidence level of 95% and a precision around the estimate of 10%, a minimum of 40 must be included. With a 20% increase to allow loss to follow-up and withdrawals during the 28-day follow-up period, a number of 50 patients should be targetted in the study per site.

8.2 Analysis of data

WHO excel programme and SPSS programme will be used for data management and analysis. Data will be analysed by two methods: the Kaplan-Meier method and per-protocol analysis. In addition to the reasons for withdrawal listed in section 3.8, patients will be considered withdrawn from the analysis if the PCR results are unclassifiable or if the results of PCR indicate that the failure is due to reinfection with *P. falciparum* or *P. vivax.*

The final analysis will include:

- a description of all patients screened and the distribution of reasons for non-inclusion in the study;
- a description of all the patients included in the study;
- the number of adverse events and serious adverse events in all the patients included in the study;
- the proportion of patients lost to follow-up or withdrawn, with 95% confidence intervals and a list of reasons for withdrawal;
- the cumulative incidence of success and failure rates at day 28, PCR-uncorrected and PCR-corrected; and
- the proportion of early treatment failure, late clinical failure, late parasitological failure and adequate clinical and parasitological response at day 28, with 95% confidence intervals, PCR-uncorrected and PCR-corrected.
- The PCT, parasite reduction rate (slope of the log-linear parasite clearance curve), the parasite reduction rate at 48 hours, and the number of patients parasite positive at day 3;
- The pharmacokinetic profile of the patients.

Guidelines on calculating the cumulative success or failure rate, the proportion of adequate clinical and parasitological response and treatment failure are given in Appendix 9.

8.3 Dissemination of results

At the end of the study, the principal investigator will submit a report on the study and the main outcome. This report will be shared with the national malaria control programme and the Ministry of health. The finding will be presented at Myanmar Research Congress and also at Vector Borne Diseases Control Program Annual Meeting.

The results will be given to study patients at each visit to study clinic. Arrangement will be made with local staff to send the results to those fail to show for follow-up. Health education talks will be given by the Principal Investigator at each supervisory visit.

8.4 Amendments to the protocol

After the protocol has been accepted, no change may be made without the agreement of the principal investigator, the sponsor(s) and the institutional review boards.

9. Ethical considerations

9.1 Approval by the national ethical committee

Before the study, official approval to conduct the study will be obtained from Institutional Ethical Committee, Department of Medical Research (Upper Myanmar) and ERC WHO.

9.2 Informed consent

Patients will be included in the study only if they give informed consent. The consent request, available in English and translated into Myanmar, will be read entirely to the patient. Details about the trial and its benefits and potential risks will be explained. Once any questions have been answered, a signature will be requested on the document (Appendix 10). If the patient is illiterate, a literate witness must sign; if possible, the signatory will be selected by the participant and will have no connection to the research team. Consent statement for the pregnancy test is also required for female participants of child-bearing age who are sexually active.

9.3 Confidentiality

All information on patients will remain confidential and be shared only by the study team. Unique identifiers will be used for computer-based data entry and blood samples. In all cases, the principal investigator will ensure that screening forms, the case report form and the completed identification code list are kept in locked files.

9.4 Health-care services

Free health care throughout follow-up for any illness related to malaria will be provided to the study patients regardless of treatment outcome; this includes any expenses related to hospital admission and to adverse medicine reactions, if required.

When prospective or actual participants are found to have diseases unrelated to malaria, the principal investigator should advise them to obtain, or refer them for, medical care.

Any subject who decides not to participate or cannot be enrolled in the study because they do not meet the criteria, will be referred to the health facility staff. They will be treated with artemether-lumefantrine and followed-up according to the standard of care established by the Ministry of Health. The principal investigator must ensure that this antimalarial drug is available at the health centre.

If a patient is withdrawn from the study before the full course of the treatment is completed, the physician must make all necessary arrangements to provide the patient with the full dose of the medicine currently tested or with a full course of artesunate (4mg/kg stat followed by 2mg/kg/day for 6 days; total dose is 16mg/kg) together with doxycycline (3mg/kg/day) or tetracycline (4mg/kg 4 times daily) also given for 7 days. If available, clindamycin (10mg/kg twice daily for 7 days) is a recommended alternative for these two groups also recommended by the national policy.

9.5 Inducement

Subjects shall be reimbursed for their transport to attend all visits to the health centre. In addition, five $US per day of hospitalization will be provided to cover the hospital fees and the transportation of the family visiting the patient.

9.6 Registration of clinical trial

As required, the study will be registered on the following website: [http://www.anzctr.org.au](http://www.ANZCTR.org.au/)

10. Budget Template

| **Human resources** |  |  |
| --- | --- | --- |
| - professional scientific staff | 5184000 |  |
| - technical staff | 6912000 |  |
| - local support | 1440000 |  |
| Sub-total | **13536000** |  |
| **Travel and transport** | 4867200 |  |
| Sub-total | **4867200** |  |
| **Equipment and supplies** |  |  |
| - equipment | 8880000 |  |
| - supplies | 3115200 |  |
| - operational costs (space rental, communication) |  |  |
| Sub-total | **11995200** |  |
| **Contingency fees for clinical trials** |  |  |
| - ethical review | 2880000 |  |
| - registration |  |  |
| - liability insurance |  |  |
| Sub-total | **2880000** |  |
| **Patient costs** | 5222400 |  |
| Sub-total | **5222400** |  |
| **Technical assistance** |  |  |
| (training, support to research institutions, |  |  |
| capacity building)(to be determined and retained by WHO) |  |  |
| Sub-total |  |  |
| **Supervision** |  |  |
| (national and consultant) | 1944000 |  |
| Sub-total | **1944000** |  |
| **Quality assurance system** |  |  |
| (data validation, slides cross-check) | 384000 |  |
| Sub-total | **384000** |  |
| **Data management** |  |  |
| (data entry, data analysis, report writing) | 192000 |  |
| Sub-total | **192000** |  |
| **Laboratory support** |  |  |
| (genotyping) | 5001600 |  |
| Sub-total | **5001600** |  |
| **Miscellaneous**  Cross border meeting )(to be determined and retained by WHO) |  |  |
| Sub-total |  |  |
|  |  |  |
| **Grand Total** | **46,022,400** |  |
|  |  |  |

11. Curriculum vitae of the principal investigator

| 1 | Family Name (surname)  First Name Myat-Phone-Kyaw Other Names |
| --- | --- |
| 2 | Date and place of birth 1 December 1955, Yangon |
| 3 | at birth: Myanmar  Nationality  at present: Myanmar |
| 4 | Academic qualifications and dates  Bachelor of Medicine and Bachelor of Surgery, MBBS 1980  Master of Medical Science (Biochemistry) (1988).  Ph. D (Malariology) (Colombo) (2004). |
| 5 | Posts held (type of post, institution/authority, dates chronologically starting with present appointment)  - Deputy Director/Head, Parasitology Research Division, DMR LM (24-4-07 to date)  - Deputy Director/Head, Experimental Medicine Research Division, DMR LM (20-12-04 to 24-4-07)  - Research Counterpart, Clinical Research Unit (Severe & Complicated Malaria), 21.1.97 to date  - Research Scientist, Parasitology Research Division, DMR LM (1. 8.98 to 20-12--04)  - Research Scientist, Clinical Research Division, DMR LM, (21.1.97 to 31.7.98)  - Senior Research Officer, Parasitology Research Division, DMR, (1-7-92 to 20.1.97)  - Assistant Lecturer, Biochemistry Department, Institute of Medicine 2, Yangon, (17-6-91 to 30-6-92)  - Demonstrator, Biochemistry Department, Institute of Medicine 2, Yangon, (11-7-83 to 16-6-91)  - Team Leader, Kayah State Sexually Transmitted Diseases and General Skin diseases Control Team  (3-5-82 to 11-7-83) |
| 6 | Publications   1. KHIN PHYU PYAR, WIN WIN MYINT, **MYAT PHONE KYAW**, THAW ZIN & MARLAR THAN (2009). Efficacy & Safety of artemisinin-piperaquine (Artequick) compared to dihydroartemisinin-piperaquine (Artekin) in uncomplicated falciparum malaria in adults. *The Myanmar Health Sciences Research Journal.,* 21 (2), 78-82. 2. Ohnmar Myint Thein, Myat Thandar, **Myat Phone Kyaw**, Maung Maung Mya, Aye Than & Ni Ni Zaw (2009). Red cell deformability and nitric oxide concentration in confirm un-complicated falciparum malaria patients. *Myanmar Medical Journal*., 52 (1), 37-40. 3. KHIN PHYU PYAR, WIN WIN MYINT, **MYAT PHONE KYAW**, THAW ZIN, KHIN NYO, THAN HTUT & MARLAR THAN (2007). Comparison of Efficacy & Safety of Five different commercial tablet formulation of oral artesunate plus mefloquine in uncomplicated falciparum malaria in adults. *The Myanmar Health Sciences Research Journal.,* 19 (2), 63-68. 4. PAING SOE, MYO KHIN, KYAW OO, **MYAT PHONE KYAW**. S. KYAW HLA, TIN TIN AUNG, AYE MAUNG HAN, NE WIN, NYUNT THEIN, SAW WIN & THAN HYEIN WIN (2007). Syringes and needles disposal by House Surgeon from major-hospitals in Yangon, Myanmar. *The Myanmar Health Sciences Research Journal.,* 19(3), 115-120. 5. WIN WIN MYINT, KHIN PHYU PYAR, **MYAT PHONE KYAW**, THAW ZIN, KHIN NYO, THAN HTUT, MARLAR THAN (2006). Comparing the efficacy of initial single dose rectal artesunate versus single dose i.v. artesunate at 24 hours, and after full consolidation treatment in both groups with intravenous artesunate in severe falciparum malaria in adults. *The Myanmar Health Sciences Research Journal.,* 18 (3), 155-160. 6. Pathirana, S.L., Alles, H.K., Bandara, S., **M. Phone-Kyaw**, Perera, M.K., Wickremasinghe, A.R., Mendis, K.N. & Handunnetti, S.M. (2005). ABO-blood-group types and protection against severe, *Plasmodium falciparum* malaria. *Annals of Tropical Medicine & Parasitology*, 99(2), 119-124. 7. SEAQUAMAT Group (2005) Artesunate versus quinine for treatment of severe falciparum malaria: a multicentre ramdomized trial South East Asian Quinine Artesunate Malaria Trial, Lancet 2005;366:717-25) 8. Ye Htut, Kyin Hla Aye, Kay Thwe Han, **Myat Phone Kyaw**, Kuino Shimono and Shingeru (2002). Feasibility and limitations of Acridine Orange Fluoresence Technique Using a Malaria diagnosis Microscope in Myanmar. *Acta Med. Okayama, 56(5), 219-222.* 9. Malar-Than, Zay-Soe, Aung-Zaw-Oo, Aye-Yu-Soe, THIN-YIN-YIN, Ohn-Ngwe, Kyi-Kyi-Tin, Khin-Nyo & **Myat-Phone-Kyaw** (2001). A prospective study to look for neuropsychiatric adverse effects related to mefloquine given as prophylaxis or as a therapeutic agent in uncomplicated malaria. *The Myanmar Health Sciences Research Journal.,* 13(1-3), 10-14. 10. Myint Oo, Marlar Than & **Myat-Phone-Kyaw** (2001). Incidence of *Plasmodium* malariae and its response to 4-Aminoquinolines. Myanmar Military Medicine 7, 6-9. 11. Malar-Than, Zay-Soe, Aung-Zaw-Oo, AYE-YU-SOE, Kyi-Kyi-Tin, Khin-Nyo & **Myat-Phone-Kyaw** (2000). A double blind comparative trial of two dosage regimens of artesunate suppository in combination with oral mefloquine in severe falciparum malaria. *The Myanmar Health Sciences Research Journal.,* 12 (1-3), 41-47. 12. **Myat-Phone-Kyaw**, Marlar-Than, Ko-Ko-Hla, Myint-Lwin, Myint-Oo & Le-Le-Win (2000). The role of blood glucose measurement in Severe and Complicated Falciparum Malaria in relation to Systemic Inflammatory Syndrome leading to Multi-organ Dysfunction. *Journal of Myanmar Military Medicine*; 6: 1-6. 13. Kay Thwe Han, Mya Thida, Ye Htut, **Myat Phone Kyaw** & Kyin Hla Aye (2000). Humoral immune response to falciparum malaria during pregnancy. *Myanmar Medical Journal.* 44(3): 120-124. 14. Myint-Oo, Marlar-Than, **Myat-Phone-Kyaw** and Tin-Shwe (2000). The use of parasite lactate dehydrogenase isoenzyme-based visual test for the speciation of *Plasmodium falciparum* and *Plasmodium vivax* infections. *Myanmar Medical Journal*; 44: 13-16. 15. Myint-Oo, Myint-Lwin, Tin-Oo, Ye-Htut, **Myat-Phone Kyaw**, Nwe-Nwe-Oo and Kyin-Hla-Aye (2000). Radiometric assessment on *in vitro* antimalarial sensitivities of *Plasmodium falciparum* isolates from Myanmar. *Myanmar Medical Journal*; 44 (2): 57-61. 16. MYINT-LWIN, HTEIN-LIN, NAY-LIN, **MYAT-PHONE-KYAW**, MYINT-OHN, NAY-SOE-MAUNG, KYAW-SOE & TIN-OO (1997). The use of personal protective measures in control of malaria in a defined community. *Southeast Asian Journal of tropical Medicine and Public Health*, 28(2): 254-259. 17. MYINT-LWIN, SOE-AUNG, **MYAT-PHONE-KYAW**, HTIN-AUNG, NYAN-SINT,YE-WIN, HLA-OO, HLA-THEIN, SOE-MYAT-TUN (1997). A simplified *in vivo* drug sensitivity test for malaria in the field. *Southeast Asian Journal of tropical Medicine and Public Health*, 28 (2): 247-253. 18. MARLAR-THAN, **MYAT-PHONE-KYAW**, AYE-YU-SOE, KHAING-KHAING-GYI, MA-SABAI & MYINT-OO (1994). Development of resistance to chloroquine by *Plasmodium vivax* infection in Myanmar. *Trans. Royl. Soc. Trop. Med. Hyg.* 89: 307-308. 19. THIN-THIN-HLAING, MYO-WIN, KHIN-MAUNG-MAUNG, **MYAT-PHONE-KYAW**, MAUNG-MAUNG-AYE, KYU-KYU-MAUNG, NYUNT-TIN & BA-AYE (1994). “A study of lipid profile in Myanmar smokers and non-smokers”. *Myanmar Health Sciences Research Journal*, 6(1): 1-3. 20. **MYAT-PHONE-KYAW**, MYINT-OO, AUNG-NAING & AYE-LWIN-HTWE (1994)." The use of primaquine in malaria infected patients with red cell glucose-6-phosphate-dehydrogenase (G6PD) enzyme deficiency in Myanmar". *Southeast Asian Journal of tropical Medicine and Public Health*, 25(4): 710-713. 21. **MYAT-PHONE-KYAW**, MYINT-OO & KYIN-HLA-AYE (1993)."*Plasmodium falciparum*: merozoite invasion rate in erythrocytes with B-thalassemia trait and glucose-6-phosphate-dehydrogenase deficient genes".  *Myanmar Health Sciences Research Journal*,5(1): 13-18. 22. **MYAT-PHONE-KYAW**, MYINT-OO, MYINT-LWIN, THAW-ZIN, KYIN-HLA-AYE & NWE-NWE-YIN (1993). "Emergence of chloroquine-resistant *Plasmodium vivax* in Myanmar". *Transactions of the Royal Society of Tropical Medicine and Hygiene*, 87:(6):687. 23. MYINT-OO, **MYAT-PHONE-KYAW** & THEIN-THAN (1990). "Some variation in membrane proteins of erythrocytes obtained from subjects with Thalassemia Trait and G-6-PD deficient genes". *Myanmar Health Sciences Research Journal*, 2(3):144-151. 24. **MYAT-PHONE-KYAW** (1988). "Cytoskeletal protein attern of red cells obtained from subjects with abnormal Haemoglobin and Glucose-6-phosphate dehydrogenase enzyme (G-6-PD) deficiency". M.Med.Sc.(Biochemistry) Thesis, Institute of Medicine, Mandalay, Myanmar. |

Appendix 1. Definition of severe falciparum malaria[[4]](#footnote-5)

**Severe manifestation of *P. falciparum* malaria in adults**

**Clinical manifestations**

- prostration,
- impaired consciousness,
- respiratory distress (metabolic acidosis),
- multiple convulsions,
- circulatory collapse,
- pulmonary oedema (radiological),
- abnormal bleeding,
- jaundice,
- haemoglobinurea.

**Laboratory findings**

- severe anaemia (haemoglobin < 5 g/dl, haematocrit < 15%),
- hypoglycaemia (blood glucose < 2.2 mmol/l or 40 mg/dl),
- acidosis (plasma bicarbonate < 15 mmol/l),
- hyperlactataemia (venous lactic acid > 5 mmol/l),
- hyperparasitaemia (> 4% in non-immune patients),
- renal impairment (serum creatinine above normal range for age).

Appendix 2. Medications (with antimalarial activity) that should not be used during the study period

- chloroquine, amodiaquine;
- quinine, quinidine;
- mefloquine, halofantrine, lumefantrine;
- artemisinin and its derivatives (artemether, arteether, artesunate, dihydroartemisinin);
- proguanil, chlorproguanil, pyrimethamine;
- sulfadoxine, sulfalene, sulfamethoxazole, dapsone;
- primaquine;
- atovaquone;
- antibiotics: tetracycline*, doxycycline, erythromycin, azythromycin, clindamycin, rifampicin, trimethoprim;
- pentamidine.

* Tetracycline eye ointments can be used.

Appendix 3. Dosing chart of ARtesunAte

Tablets containing 50 mg of artesunate. Doses will be rounded to the nearest quarter-tablet. The dose in mg and in # tablets will be recorded in the CRF Dose Administration Form. These are the same doses as used in the ‘Clinical investigation of in-vivo susceptibility of *P. falciparum* to artesunate in Western Cambodia’

| **Weight (kg)** | **Artesunate** | |
| --- | --- | --- |
|  | **4 mg/kg (OD)** | |
|  | Mg | **Tablets** (50mg) |
| **30-32** | 125 | **2 1/2** |
| **33-34** | 137.5 | **2 3/4** |
| **35** | 137.5 | **2 3/4** |
| **36-39** | 150 | **3** |
| **40** | 162.5 | **3 1/4** |
| **41-42** | 162.5 | **3 1/4** |
| **43-45** | 175 | **3 1/2** |
| **46** | 187.5 | **3 3/4** |
| **47-48** | 187.5 | **3 3/4** |
| **49-51** | 200 | **4** |
| **52-53** | 212.5 | **4 1/4** |
| **54** | 212.5 | **4 1/4** |
| **55-57** | 225 | **4 1/2** |
| **58-59** | 237.5 | **4 3/4** |
| **60** | 237.5 | **4 3/4** |
| **61-64** | 250 | **5** |
| **65** | 262.5 | **5 1/4** |
| **66-67** | 262.5 | **5 1/4** |
| **68-70** | 275 | **5 1/2** |

Appendix 4. Classification of treatment outcomes[[5]](#footnote-6)

**Early treatment failure**

- danger signs or severe malaria on day 1, 2 or 3 in the presence of parasitaemia;
- parasitaemia on day 2 higher than on day 0, irrespective of axillary temperature;
- parasitaemia on day 3 with axillary temperature ≥ 37.5 ºC;
- parasitaemia on day 3 ≥ 25% of count on day 0.

**Late treatment failure**

**Late clinical failure**

- danger signs or severe malaria in the presence of parasitaemia on any day between day 4 and day 28 (day 42) in patients who did not previously meet any of the criteria of early treatment failure;
- presence of parasitaemia on any day between day 4 and day 28 with axillary temperature
  ≥ 37.5 ºC (or history of fever) in patients who did not previously meet any of the criteria of early treatment failure

**Late parasitological failure**

- presence of parasitaemia on any day between day 7 and day 28 with axillary temperature
  < 37.5 ºC in patients who did not previously meet any of the criteria of early treatment failure or late clinical failure

**Adequate clinical and parasitological response**

- absence of parasitaemia on day 28, irrespective of axillary temperature, in patients who did not previously meet any of the criteria of early treatment failure, late clinical failure or late parasitological failure

Appendix 5. Case screening form

**.**

**.**

| **Case screening form** | | | | | |
| --- | --- | --- | --- | --- | --- |
| Health centre name: | | | | | Study number: |
| Locality: | | | | | Patient screening number: |
| District: | | | | | Date of visit (dd-mmm-yyyy): |
| Province: | | | | |  |
| **Demographic data** | | | | | |
| Date of birth (dd-mmm-yyyy): | | | or estimated age:       in:  months or  years | | |
| Height (cm): | Weight (kg): | |  | | |
| Sex:  Male  Female | | |
| If female, is the patient pregnant?  Yes  No  Not sure | | | | | |
| If pregnant, provide the date of the last menstrual period (dd-mmm-yyyy): | | | | | |
| **Pre-treatment temperature** | | | | | |
| History of fever in previous 24 h?  Yes  No | | | | | |
| Temperature:       ºC  Axillary  Tympanic  Rectal  Oral | | | | | |
| **Thick and thin blood smears for estimation of *P. falciparum*** **parasite counts** | | | | | |
| Species:  *P. falciparum*  *P. vivax*  *P. ovale*  *P. malariae* | | | | | |
| Were species other than *P. falciparum* present?  Yes  No **(If yes, patient is not eligible).** | | | | | |
| Approximate number of *P. falciparum* asexual parasites:  Presence of 10–100 parasites / 6 white blood cells?  Yes  No **(If no, patient is not eligible)** | | | | | |
| Presence of *P. falciparum* gametocytes?  Yes  No | | | | | |
| Has a blood sample for PCR been collected?  Yes  No | | | | | |
| Haemoglobin:       g/dl | | Haematocrit:       % | | | |
| **Urinary analysis (pregnancy test for female patients)** | | | | | |
| Result of pregnancy test:  Positive  Negative **(If positive, patient is not eligible)** | | | | | |
| **Inclusion criteria** | | | | | |
| - age > 18years - mono-infection with *P. falciparum* confirmed by positive blood smear (i.e. no mixed infection) - parasitaemia between 10000 and 100000/µl of asexual forms - measured temperature (depending on method of measurement) or history of fever within previous 24 h - ability to swallow oral medication - ability and willingness to comply with the study protocol for the duration of the study and to comply with the study visit schedule - absence of severe malnutrition (defined as per protocol) | | | | | |
| Does the patient meet all the inclusion criteria?  Yes  No **(If no, patient is not eligible)** | | | | | |
| **Case screening form (page 2)** | | | | | |
| **Exclusion criteria** | | | | | |
| - signs and symptoms of severe or complicated malaria requiring parenteral treatment according to WHO criteria (Appendix 1) - mixed or mono-infection with another *Plasmodium* species detected by microscopy - severe malnutrition - febrile conditions caused by diseases other than malaria or other known underlying chronic or severe diseases - regular medication which interferes with antimalarial pharmacokinetics - history of hypersensitivity reactions or contraindications to the medicine tested - positive pregnancy test or breastfeeding - unable to or unwilling to take contraceptives. | | | | | |
| Does the patient meet any of the exclusion criteria?  Yes  No **(If yes, the patient is not eligible)** | | | | | |
| If yes, please specify the reason for exclusion: | | | | | |
| **Patient informed consent and assent** | | | | | |
| Consent form signed:  Yes  No | | | | Patient identity number:  Date (dd-mmm-yyyy): | |
| Assent form signed:  Yes  No | | | |

Appendix 6. Schedule of follow-up activities

|  | Day | | | | | | | | | |
| --- | --- | --- | --- | --- | --- | --- | --- | --- | --- | --- |
|  | 0 | 1 | 2 | 3 | 7 | 14 | 21 | 28 | Any other |  |
| **Procedure** |  |  |  |  |  |  |  |  |  |  |
| Clinical assessment | X | X | X | X | X | X | X | X | (X) |  |
| Temperature | X | X | X | X | X | X | X | X | (X) |  |
| Blood slide for parasite count | X |  | X | X | X | X | X | X | (X) |  |
| Urine sample | (X) |  |  |  |  |  |  |  |  |  |
| Blood for:  genotyping  molecular markers  antimalarial blood concentration | X  X  11 X | X |  |  | X | X | X | X | X |  |
| Treatment |  |  |  |  |  |  |  |  |  |  |
| Medicine to be tested from day 0 to day 6 included | X | X | X | X |  |  |  |  |  |  |
| Rescue treatment |  | (X) | (X) | (X) | (X) | (X) | (X) | (X) | (X) |  |

Parentheses denote conditional or optional activities. For example, treatment would be given on days 1 and 2 only for 3-day dosing. On day 1, the patient should be examined for parasitaemia if he or she has any danger signs. Rescue treatment could be given on any day, provided that the patient meets the criteria for treatment failure. Extra days are any days other than regularly scheduled follow-up days when the patient returns to the facility because of recurrence of symptoms. On extra days, blood slides may be taken routinely or at the request of the clinical staff.

**Day 0**

**Screening**

- clinical assessment, including measurement of weight and height; referral in cases of severe malaria or danger signs;
- measurement of temperature;
- parasitological assessment;
- pregnancy test (if necessary);
- informed consent.

**Enrolment**

- treatment, first dose;
- blood sampling for genotyping.

**Optional**

- urinary test to detect antimalarial drugs;
- haemoglobin/haematocrit;
- molecular markers of drug resistance;
- in vitro test;
- antimalarial drug blood concentration.

**Day 1**

- clinical assessment; referral in cases of severe malaria or danger signs;
- measurement of axillary temperature;
- parasitological assessment in cases of severe malaria or danger signs;
- treatment, second dose or alternative treatment in case of early treatment failure.

**Day 2, Day 3**

- clinical assessment; referral in cases of severe malaria or danger signs;
- measurement of axillary temperature;
- parasitological assessment;
- treatment, third to seventh dose (until day 6) or alternative treatment in case of early treatment failure.

**Day 7, Day 14, day 21, day 28, any other day**

- clinical assessment; referral in cases of severe malaria or danger signs;
- measurement of axillary temperature;
- parasitological assessment;
- alternative treatment in cases of treatment failure;
- pregnancy test at the end of follow-up (if necessary);
- blood sampling for genotyping to distinguish between recrudescence and reinfection in cases of treatment failure after day 7.

**Optional (after day 7)**

- haemoglobin/haematocrit;
- blood sampling for molecular markers for drug resistance, antimalarial blood concentration.

Appendix 7. Case report forms

| **Case report form: follow-up day 0** | | | | | | | | | | |
| --- | --- | --- | --- | --- | --- | --- | --- | --- | --- | --- |
| Health centre name: | | | | | | | | Study number: | | |
| Locality: | | | | | | | | Patient identity number: | | |
| District: | | | | | | | | Date of visit (dd-mmm-yyyy): | | |
| Province: | | | | | | | |  | | |
| **Demographic data** | | | | | | | | | | |
| Date of birth (dd-mmm-yyyy): | | | | | or estimated age:       in:  months or  years | | | | | |
| Height (cm): | | Weight (kg): | | | Sex:  Male  Female | | | | | |
| If female, is the patient pregnant?  Yes  No  Not sure **(If yes, patient is not eligible).** | | | | | | | | | | |
| If pregnant, provide the date of the last menstrual period (dd-mmm-yyyy): | | | | | | | | | | |
| **Pre-treatment temperature** | | | | | | | | | | |
| History of fever in previous 24 h?  Yes  No | | | | | | | | | | |
| Temperature:       ºC  Axillary  Tympanic  Rectal  Oral | | | | | | | | | | |
| **Thick blood smears for** ***P. falciparum*: quantitative parasite counts and qualitative gametocyte counts** | | | | | | | | | | |
| Average number of asexual *P. falciparum* parasites/μl at Day 0 H0: | | | | | | | Average number of asexual *P. falciparum* parasites/μl at Day 0 H12: | | | |
| Presence of *P. falciparum* gametocytes at Day 0 H0?  Yes  No | | | | | | | Presence of *P. falciparum* gametocytes at Day 0 H0?  Yes  No | | | |
| Were species other than *P. falciparum* present?  Yes  No **(If yes, patient is not eligible).** | | | | | | | | | | |
| If yes, which species?  *P. vivax*  *P. ovale*  *P. malariae* | | | | | | | | | | |
| Has blood sample for PCR been collected?  Yes  No | | | | | | | | | | |
| **Urinary test for antimalarial drugs** | | | | | | | | | | |
| Test used: | | | | Test result:  Positive  Negative | | | | | | |
| **Prior medication** | | | | | | | | | | |
| All prior medication, including natural remedies and homeopathic medicines, taken within the previous 14 days should be reported in this section. | | | | | | | | | | |
| Has the patient taken any prior antimalarial medication?  Yes  No. If yes, please specify below. Either the date of stopping or the ‘ongoing’ box should be checked. | | | | | | | | | | |
| Medicine name (generic name) | Dates | | Ongoing  (Yes = ) | | | Total daily dose and unit (e.g. 400 mg) | | | Route of administration | Indication for use |
|  | Start: | |  | | |  | | |  |  |
|  | Stop: | |  |  |  |  |  |  |  |  |
|  | Start: | |  | | |  | | |  |  |
|  | Stop: | |  |  |  |  |  |  |  |  |
|  | Start: | |  | | |  | | |  |  |
|  | Stop: | |  |  |  |  |  |  |  |  |

| **Case report form: follow-up day 0 (page 2)** | | | | | |
| --- | --- | --- | --- | --- | --- |
| **Medication administration** | | | | | |
| Name(s) of antimalarial drug(s) | | Time of dose (hh:min) | Number of tablets | Did the patient vomit? | Time of vomiting (hh:min) |
|  | |  |  | Yes  No |  |
|  | |  |  | Yes  No |  |
| Name(s) of other medicine(s) |  | | | | |
|  | |  |  | Yes  No |  |
|  | |  |  | Yes  No |  |

| **Case report form: follow-up day 1** | | | | | |
| --- | --- | --- | --- | --- | --- |
| Study number: | | | | | |
| Patient identity number: | | | | | |
| Date of visit (dd-mmm-yyyy): | | | | | |
| **Clinical status** | | | | | |
| Presence of danger signs or signs of severe or complicated malaria?  Yes  No  If yes, perform thick blood smear. | | | | | |
| Temperature:       ºC  Axillary  Tympanic  Rectal  Oral | | | | | |
| **Thick blood smears for estimation of *P. falciparum* parasite counts** | | | | | |
| Average number of asexual *P. falciparum* parasites/μl at Day 1 H0: | | | Average number of asexual *P. falciparum* parasites/μl at Day 1 H12: | | |
| Presence of *P. falciparum* gametocytes at Day 1 H0?  Yes  No | | | Presence of *P. falciparum* gametocytes at Day 1 H12?  Yes  No | | |
| Were species other than *P. falciparum* present?  Yes  No | | | | | |
| If yes, which species?  *P. vivax*  *P. ovale*  *P. malariae* | | | | | |
| **Adverse events** | | | | | |
| Presence of an adverse event?  Yes  No | | | | | |
| If yes, name the adverse event: | | | | | |
| Is it a serious adverse event?  Yes  No. If yes, inform the sponsor. | | | | | |
| **Medication administration** | | | | | |
| Name(s) of antimalarial drug(s) | Time of dose (hh:min) | Number of tablets | | Did the patient vomit? | Time of vomiting (hh:min) |
|  |  |  | | Yes  No |  |
|  |  |  | | Yes  No |  |
| Name(s) of other medicine(s) |  | | | | |
|  |  |  | | Yes  No |  |
|  |  |  | | Yes  No |  |

| **Case report form: follow-up day 2** | | | | | |
| --- | --- | --- | --- | --- | --- |
| Study number: | | | | | |
| Patient identity number: | | | | | |
| Date of visit (dd-mmm-yyyy): | | | | | |
| **Clinical status** | | | | | |
| Presence of danger signs or signs of severe or complicated malaria?  Yes  No | | | | | |
| Temperature:       ºC  Axillary  Tympanic  Rectal  Oral | | | | | |
| **Thick blood smears for estimation of *P. falciparum* parasite counts** | | | | | |
| Average number of asexual *P. falciparum* parasites/μl at Day 2 H0: | | | Average number of asexual *P. falciparum* parasites/μl at Day 2 H12: | | |
| Presence of *P. falciparum* gametocytes at Day 2 H0?  Yes  No | | | Presence of *P. falciparum* gametocytes at Day 2 H12?  Yes  No | | |
| Were species other than *P. falciparum* present?  Yes  No | | | | | |
| If yes, which species?  *P. vivax*  *P. ovale*  *P. malariae* | | | | | |
| **Adverse events** | | | | | |
| Presence of an adverse event?  Yes  No | | | | | |
| If yes, name the adverse event: | | | | | |
| Is it a serious adverse event?  Yes  No. If yes, inform the sponsor. | | | | | |
| **Medication administration** | | | | | |
| Name(s) of antimalarial drug(s) | Time of dose (hh:min) | Number of tablets | | Did the patient vomit? | Time of vomiting (hh:min) |
|  |  |  | | Yes  No |  |
|  |  |  | | Yes  No |  |
| Name(s) of other medicine(s) |  | | | | |
|  |  |  | | Yes  No |  |
|  |  |  | | Yes  No |  |

| **Case report form:** **follow-up day 3** | | | | | |
| --- | --- | --- | --- | --- | --- |
| Study number: | | | | | |
| Patient identity number: | | | | | |
| Date of visit (dd-mmm-yyyy): | | | | | |
| **Clinical status** | | | | | |
| Presence of danger signs or signs of severe or complicated malaria?  Yes  No | | | | | |
| Temperature:       ºC  Axillary  Tympanic  Rectal  Oral | | | | | |
| **Thick blood smears for estimation of** ***P. falciparum* parasite counts** | | | | | |
| Average number of asexual *P. falciparum* parasites/μl at Day 3 H0: | | | Average number of asexual *P. falciparum* parasites/μl at Day 3 H12: | | |
| Presence of *P. falciparum* gametocytes at Day 3 H0?  Yes  No | | | Presence of *P. falciparum* gametocytes at Day 3 H12?  Yes  No | | |
| Were species other than *P. falciparum* present?  Yes  No | | | | | |
| If yes, which species?  *P. vivax*  *P. ovale*  *P. malariae* | | | | | |
| **Adverse events** | | | | | |
| Presence of an adverse event?  Yes  No | | | | | |
| If yes, name the adverse event: | | | | | |
| Is it a serious adverse event?  Yes  No. If yes, inform the sponsor. | | | | | |
| **Medication administration** | | | | | |
| Name(s) of antimalarial drug(s) | Time of dose (hh:min) | Number of tablets | | Did the patient vomit? | Time of vomiting (hh:min) |
|  |  |  | | Yes  No |  |
|  |  |  | | Yes  No |  |
| Name(s) of other medicine(s) |  | | | | |
|  |  |  | | Yes  No |  |
|  |  |  | | Yes  No |  |

| **Case report form: follow-up day 7** | | | | | |
| --- | --- | --- | --- | --- | --- |
| Study number: | | | | | |
| Patient identity number: | | | | | |
| Date of visit (dd-mmm-yyyy): | | | | | |
| **Clinical status** | | | | | |
| Presence of danger signs or signs of severe or complicated malaria?  Yes  No | | | | | |
| History of fever within previous 24 h?  Yes  No | | | | | |
| Temperature:       ºC  Axillary  Tympanic  Rectal  Oral | | | | | |
| **Thick blood smears for estimation of *P. falciparum* parasite counts** | | | | | |
| Average number of asexual *P. falciparum* parasites/μl: | | | | | |
| Presence of *P. falciparum* gametocytes?  Yes  No | | | | | |
| Were species other than *P. falciparum* present?  Yes  No | | | | | |
| If yes, which species?  *P. vivax*  *P. ovale*  *P. malariae* | | | | | |
| Has a blood sample for PCR been collected?  Yes  No | | | | | |
| **Adverse events** | | | | | |
| Presence of an adverse event?  Yes  No | | | | | |
| If yes, name the adverse event: | | | | | |
| Is it a serious adverse event?  Yes  No. If yes, inform the sponsor. | | | | | |
| **Medication administration** | | | | | |
| Name(s) of antimalarial drug(s) | | Time of dose (hh:min) | Number of tablets | Did the patient vomit? | Time of vomiting (hh:min) |
|  | |  |  | Yes  No |  |
|  | |  |  | Yes  No |  |
| Name(s) of other medicine(s) |  | | | | |
|  | |  |  | Yes  No |  |
|  | |  |  | Yes  No |  |

| **Case report form: follow-up day 14** | | | | |
| --- | --- | --- | --- | --- |
| Study number: | | | | |
| Patient identity number: | | | | |
| Date of visit (dd-mmm-yyyy): | | | | |
| **Clinical status** | | | | |
| Presence of danger signs or signs of severe or complicated malaria?  Yes  No | | | | |
| History of fever within previous 24 h?  Yes  No | | | | |
| Temperature:       ºC  Axillary  Tympanic  Rectal  Oral | | | | |
| **Thick blood smears for estimation of *P. falciparum* parasite counts** | | | | |
| Average number of asexual *P. falciparum* parasites/μl: | | | | |
| Presence of *P. falciparum* gametocytes?  Yes  No | | | | |
| Were species other than *P. falciparum* present?  Yes  No | | | | |
| If yes, which species?  *P. vivax*  *P. ovale*  *P. malariae* | | | | |
| Has a blood sample for PCR been collected?  Yes  No | | | | |
| **Adverse events** | | | | |
| Presence of an adverse event?  Yes  No | | | | |
| If yes, name the adverse event: | | | | |
| Is it a serious adverse event?  Yes  No. If yes, inform the sponsor. | | | | |
| **Medication administration** | | | | |
| Name(s) of antimalarial drug(s) | Time of dose (hh:min) | Number of tablets | Did the patient vomit? | Time of vomiting (hh:min) |
|  |  |  | Yes  No |  |
|  |  |  | Yes  No |  |
| Name(s) of other medicine(s) |  | | | |
|  |  |  | Yes  No |  |
|  |  |  | Yes  No |  |

| **Case report form: follow-up day 21** | | | | |
| --- | --- | --- | --- | --- |
| Study number: | | | | |
| Patient identity number: | | | | |
| Date of visit (dd-mmm-yyyy): | | | | |
| **Clinical status** | | | | |
| Presence of danger signs or signs of severe or complicated malaria?  Yes  No | | | | |
| History of fever within previous 24 h?  Yes  No | | | | |
| Temperature:       ºC  Axillary  Tympanic  Rectal  Oral | | | | |
| **Thick blood smears for estimation of *P. falciparum* parasite counts** | | | | |
| Average number of asexual *P. falciparum* parasites/μl: | | | | |
| Presence of *P. falciparum* gametocytes?  Yes  No | | | | |
| Were species other than *P. falciparum* present?  Yes  No | | | | |
| If yes, which species?  *P. vivax*  *P. ovale*  *P. malariae* | | | | |
| Has a blood sample for PCR been collected?  Yes  No | | | | |
| **Adverse events** | | | | |
| Presence of an adverse event?  Yes  No | | | | |
| If yes, name the adverse event: | | | | |
| Is it a serious adverse event?  Yes  No. If yes, inform the sponsor. | | | | |
| **Medication administration** | | | | |
| Name(s) of antimalarial drug(s) | Time of dose (hh:min) | Number of tablets | Did the patient vomit? | Time of vomiting (hh:min) |
|  |  |  | Yes  No |  |
|  |  |  | Yes  No |  |
| Name(s) of other medicine(s) |  | | | |
|  |  |  | Yes  No |  |
|  |  |  | Yes  No |  |

| **Case report form: day ___( any other day that is not part of regular follow-up)** | | | | |
| --- | --- | --- | --- | --- |
| Study number: | | | | |
| Patient identity number: | | | | |
| Date of visit (dd-mmm-yyyy): | | | | |
| **Clinical status** | | | | |
| Presence of danger signs or signs of severe or complicated malaria?  Yes  No | | | | |
| History of fever within previous 24 h?  Yes  No | | | | |
| Temperature:       ºC  Axillary  Tympanic  Rectal  Oral | | | | |
| **Thick blood smears for estimation of *P. falciparum*** **parasite counts** | | | | |
| Average number of asexual *P. falciparum* parasites/μl: | | | | |
| Presence of *P. falciparum* gametocytes?  Yes  No | | | | |
| Were species other than *P. falciparum* present?  Yes  No | | | | |
| If yes, which species?  *P. vivax*  *P. ovale*  *P. malariae* | | | | |
| Has a blood sample for PCR been collected?  Yes  No | | | | |
| **Adverse events** | | | | |
| Presence of an adverse event?  Yes  No | | | | |
| If yes, name the adverse event: | | | | |
| Is it a serious adverse event?  Yes  No. If yes, inform the sponsor. | | | | |
| **Medication administration** | | | | |
| Name(s) of antimalarial drug(s) | Time of dose (hh:min) | Number of tablets | Did the patient vomit? | Time of vomiting (hh:min) |
|  |  |  | Yes  No |  |
|  |  |  | Yes  No |  |
| Name(s) of other medicine(s) |  | | | |
|  |  |  | Yes  No |  |
|  |  |  | Yes  No |  |

| **Case report form: follow-up day 35** | | | | |
| --- | --- | --- | --- | --- |
| Study number: | | | | |
| Patient identity number: | | | | |
| Date of visit (dd-mmm-yyyy): | | | | |
| **Clinical status** | | | | |
| Presence of danger signs or signs of severe or complicated malaria?  Yes  No | | | | |
| History of fever within previous 24 h?  Yes  No | | | | |
| Temperature:       ºC  Axillary  Tympanic  Rectal  Oral | | | | |
| **Thick blood smears for estimation of *P. falciparum* parasite counts** | | | | |
| Average number of asexual *P. falciparum* parasites/μl: | | | | |
| Presence of *P. falciparum* gametocytes?  Yes  No | | | | |
| Were species other than *P. falciparum* present?  Yes  No | | | | |
| If yes, which species?  *P. vivax*  *P. ovale*  *P. malariae* | | | | |
| Has a blood sample for PCR been collected?  Yes  No | | | | |
| **Adverse events** | | | | |
| Presence of an adverse event?  Yes  No | | | | |
| If yes, name the adverse event: | | | | |
| Is it a serious adverse event?  Yes  No. If yes, inform the sponsor. | | | | |
| **Medication administration** | | | | |
| Name(s) of antimalarial drug(s) | Time of dose (hh:min) | Number of tablets | Did the patient vomit? | Time of vomiting (hh:min) |
|  |  |  | Yes  No |  |
|  |  |  | Yes  No |  |
| Name(s) of other medicine(s) |  | | | |
|  |  |  | Yes  No |  |
|  |  |  | Yes  No |  |

| **Case report form: final day of follow-up (28/42)** | | | | | | | |
| --- | --- | --- | --- | --- | --- | --- | --- |
| Study number: | | | | | | | |
| Patient identity number: | | | | | | | |
| Date of visit (dd-mmm-yyyy): | | | | | | | |
| **Clinical status** | | | | | | | |
| Presence of danger signs or signs of severe or complicated malaria?  Yes  No | | | | | | | |
| History of fever within previous 24 h?  Yes  No | | | | | | | |
| Temperature:       ºC  Axillary  Tympanic  Rectal  Oral | | | | | | | |
| **Thick blood smears for estimation of *P****.* ***falciparum* parasite counts** | | | | | | | |
| Average number of asexual *P. falciparum* parasites/μl: | | | | | | | |
| Presence of *P. falciparum* gametocytes?  Yes  No | | | | | | | |
| Were species other than *P. falciparum* present?  Yes  No | | | | | | | |
| If yes, which species?  *P. vivax*  *P. ovale*  *P. malariae* | | | | | | | |
| Has a blood sample for PCR been collected?  Yes  No | | | | | | | |
| **Adverse events** | | | | | | | |
| Presence of an adverse event?  Yes  No | | | | | | | |
| If yes, name the adverse event: | | | | | | | |
| Is it a serious adverse event?  Yes  No. If yes, inform the sponsor. | | | | | | | |
| **Medication administration** | | | | | | | |
| Name(s) of antimalarial drug(s) | Time of dose (hh:min) | | Number of tablets | | Did the patient vomit? | Time of vomiting (hh:min) | |
|  |  | |  | | Yes  No |  | |
|  |  | |  | | Yes  No |  | |
| Name(s) of other medicine(s) |  | | | | | | |
|  |  |  | | | Yes  No |  | |
|  |  |  | | | Yes  No |  | |
| **Urinary analysis (pregnancy test for female patients)** | | | | | | | |
| **Patients with a positive pregnancy test must be followed up for 6–8 weeks after delivery** | | | | | | | |
| Result of pregnancy test:  Positive  Negative | | | | Date of test (dd-mmm-yyyy): | | | |
| If the patient is pregnant, follow-up of the pregnancy is required, including: clinical examination of the infant at birth and 6-8 weeks after birth. Please provide comments below. If needed fill in the serious adverse event report form: | | | | | | | |
| **Case report form: final day of follow-up (28/42) (page 2)** | | | | | | | |
| **Overall assessment** | | | | | | | |
| Outcome:  adequate clinical and parasitological response  early treatment failure  late clinical failure  late parasitological failure  lost to follow-up  withdrawn | | | | | | | |
| Outcome occurred on follow-up day:      (e.g. 1, 2, 3, 7, 14, …) | | | | | | | |
| PCR:  *P. falciparum* recrudescence  *P. falciparum* reinfection  other species  mixed with *P. falciparum* recrudescence  mixed with *P. falciparum* reinfection  unknown | | | | | | |  |
| PCR corrected results:  adequate clinical and parasitological response  early treatment failure  late clinical failure  late parasitological failure  lost to follow-up  withdrawn | | | | | | |  |
| Reason for withdrawal: | | | | | | | |
| Other comments: | | | | | | | |

Appendix 8. Serious adverse event report form

| **Serious adverse event report form** | | | |
| --- | --- | --- | --- |
| Health centre name: | | | Study number: |
| Locality: | | | Patient identity number: |
| District: | | | Date of visit (dd-mmm-yyyy): |
| Province: | | | Follow-up day: |
| **Demographic data** | | | |
| Date of birth (dd-mmm-yyyy): | | or estimated age:       in:  months or  years | |
| Height (cm): | Weight (kg): |  | |
| Sex:  Male  Female | |
| If female, is the patient pregnant?  Yes  No  Not sure | | | |
| If pregnant, provide the date of the last menstrual period (dd-mmm-yyyy): | | | |
| **Serious adverse event** | | | |
| Type of event: | | | |
| Death | | | |
| Life-threatening | | | |
| Hospitalization or prolongation of hospitalization | | | |
| Permanent disability | | | |
| Congenital anomaly or birth defect  Date of occurrence (dd-mmm-yyyy): | | | |
| Describe the serious adverse event (include all relevant laboratory results): | | | |
| Describe how the reaction was treated: | | | |

| **Serious adverse event report form (page 2)** | | | | | | | |
| --- | --- | --- | --- | --- | --- | --- | --- |
| Comments (e.g. relevant medical history, drug allergies, previous exposure to similar drugs, other laboratory data, whether reaction abated after stopping the drug, whether reaction reappeared after reintroduction): | | | | | | | |
| **Outcome** | | | | | | | |
| Recovered completely | | | | | | | |
| Not yet recovered | | | | | | | |
| Recovered with long-term consequences  If patient recovered, provide date of recovery (dd-mmm-yyyy): | | | | | | | |
| **Medicines** (list the **medicine suspected of causing** the serious adverse event as well as all **concomitant medicines**) | | | | | | | |
| Brand name, batch number, manufacturer name  (list suspected medicine first) | Daily dose | Route | | | Start date | End date | Indications for use |
|  |  |  | | |  |  |  |
|  |  |  | | |  |  |  |
|  |  |  | | |  |  |  |
|  |  |  | | |  |  |  |
|  |  |  | | |  |  |  |
| **Reporting officer** | | | | | | | |
| Name: | | | | | | | |
| Qualification: | | | | | | | |
| Address: | | | | | | | |
| Phone: | | |  | | | | |
| Fax: | | |  | | | | |
| Email: | | | | | | | |
| Signature: | | | | Date: | | | |

Appendix 9. Guidelines for analysis of results

| **End-point for day X**  **(X = 28)** | **PCR-uncorrected results** | |
| --- | --- | --- |
|  | **Cumulative success or failure rate (Kaplan-Meier analysis)** | **Proportion**  **(per-protocol analysis)** |
| Adequate clinical and parasitological response on day X | Success | Success |
| Early treatment failure | Failure | Failure |
| Late clinical failure before day 7 | Failure | Failure |
| Late clinical failure or late parasitological failure on or after day 7 | Failure | Failure |
| Other species infection | Censored day of infection | Excluded from analysis |
| Lost to follow-up | Censored last day of follow-up according to timetable | Excluded from analysis |
| Withdrawal and protocol violation | Censored last day of follow-up according to timetable before withdrawal or protocol violation | Excluded from analysis |

| **End-point for day X**  **(X = 28)** | **PCR-corrected results** | |
| --- | --- | --- |
|  | **Cumulative success or failure rate (Kaplan-Meier analysis)** | **Proportion**  **(per-protocol analysis)** |
| Adequate clinical and parasitological response at day X | Success | Success |
| Early treatment failure | Failure | Failure |
| Late clinical failure before day 7 | Failure | Failure |
| Late clinical failure or late parasitological failure on or after day 7 |  |  |
| - falciparum recrudescence* | Failure | Failure |
| - falciparum reinfection* | Censored day of reinfection | Excluded from analysis |
| - other species mixed with falciparum recrudescence | Failure | Failure |
| - other species mixed with falciparum reinfection | Censored day of reinfection | Excluded from analysis |
| - other species infection | Censored day of infection | Excluded from analysis |
| - undetermined or missing PCR | Excluded from analysis | Excluded from analysis |
| Lost to follow-up | Censored last day of follow-up according to timetable | Excluded from analysis |
| Withdrawal and protocol violation | Censored last day of follow-up according to timetable before protocol violation or withdrawal | Excluded from analysis |

* WHO. *Methods and techniques for clinical trials on antimalarial drug efficacy: genotyping to identify parasite populations*.
Geneva, World Health Organization, 2008 (<http://www.who.int/malaria/resistance>).

Appendix 10. Consent form[[6]](#footnote-7)

**Example of an informed consent form for adults**

This informed consent form is for adults 18 years of age inclusive who attend Kawthaung (Tanintharyi Division) who have been invited to participate in a study to evaluate the efficacy of artesunate for the treatment of uncomplicated falciparum malaria.

| Name of principal investigator: |  | Dr. Myat-Phone-Kyaw |
| --- | --- | --- |
| Name of organization: |  | Department of Medical Research (Lower Myanmar |
| Name of sponsor: |  | Department of Medical Research (Lower Myanmar |
| Name of proposal and version: |  | Myanmar 2/2010 v 1 |

This informed consent form has two parts:

1. Information sheet (to share information about the study with you)
2. Certificate of consent (for signatures if you agree to take part)

You will be given a copy of the full informed consent form.

**Part I. Information sheet**

My name is Dr Myat-Phone-Kyaw, and I work for the Ministry of Health. We are doing a study on the treatment of malaria. Malaria is a dangerous disease; however, it can be treated with medicine. The purpose of this study is to confirm that the medicine, called artesunate, is still effective for curing malaria.

We are inviting 40 adults living in this area to take part in this study.

I am going to give you information and invite you to participate in this surveillance study. Before you decide whether to participate, you can talk to anyone you feel comfortable with. There may be some words that you do not understand. Please ask me to stop as we go through the information, and I will take time to explain. If you have questions later, you can ask me, the study doctor or the staff.

Your participation in this study is entirely voluntary. It is your choice whether to participate or not. Whether you choose to participate or not, all the services you receive at this clinic will continue and nothing will change. If you choose not to participate in this project, we will offer the treatment that is routinely provided in this clinic for malaria, and we will tell you more about it later. You may change your mind later and stop participating even if you agreed earlier.

You will receive 7 doses of medicine over 7 days at a dose that is considered to be safe. As the parasites that cause malaria can become resistant to the medicine, the Ministry regularly does studies to make sure the medicine is still working. The medicine mane is Arsumax; it is produced with the trade name Guilin. This medicine is known to be very effective, but you should know that it has some minor side-effects: very rarely rash and low blood cell counts can occur, but these should not have a serious effect on your health.

If we find that the medicine is not working, we will use what is called ‘rescue medicine’. This medicine is called artemether-lumefantrine and is given over 3 days. You should know that this medicine has some minor side-effects: headache, nausea, abdominal discomfort, dizziness.

The study will take place over 28 days. During that time, you will be hospitalized over 7 days and then have to come to the health facility for 1 hour each day for 3 days according to the scheduled dates given to you. At the end of 4 weeks, the study will be finished. At each visit, you will be examined by a physician.

Today, we will take 1 time 6 ml of blood (equivalent of a big spoon) and 10 times 1 ml of blood (equivalent of half a little spoon) is taken from your arm for testing. Tomorrow and after tomorrow, we will take twice two drops of blood from your finger. We will take blood from your finger twice a day until the parasites disappear from our blood. The last day of hospitalization you will have a blood test from your finger, as well as your 1^st^, 2^nd^ and 3rd visits. At the total 17.4 ml of blood will taken (equivalent to 3 big spoons).

|  | Total volume of blood sampled | | | | | | | | | |
| --- | --- | --- | --- | --- | --- | --- | --- | --- | --- | --- |
| Day | 0 | 1 | 2 | 3 | 4 | 7 | 14 | 21 | 28 | Total |
| Volume of blood (ml) | 16.2 | 0.1 | 0.1 | 0.1 | 0.1 | 0.2 | 0.2 | 0.2 | 0.2 | 17.4 |

When blood is taken from your arm or your finger, you may experience a bit of pain or fear. The pain should disappear within 1 day. The blood samples will be used to study the malaria in your blood and level of the drug in your blood. The examination of some of the blood samples will be done during or immediately after the study and it will not affect the success of the treatment. Nothing else will be done with your blood.

If you do not attend the scheduled visit, we will visit you at home.

As already mentioned, this medicine can have some minor side effects. It is also possible that it may cause some problems that we are not aware of; however, we will follow you closely and keep track of these effects, if they arise, and of any other problems. We will give you a telephone number to call if you notice anything out of the ordinary or if you have concerns or questions. You can also come to this health facility at any time and ask to see Dr. Myat Phone Kyaw, Deputy Director, Parasitology Research Division, Department of Medical Research, No 5, Ziwaka Road, Yangon 11191, Myanmar; Telephone Number 01 375447 Extension 153. If you experience side-effects, we may use some other medicine, free of charge, which will help to reduce the symptoms or reactions, or we may stop one or more of the medicines. If this is necessary we will discuss it together. You will always be consulted before we move to the next step.

If you decide to participate in this study, any illnesses related to malaria or to the malaria treatment will be treated at no charge to you. Your participation will help us to make sure the medicine is still working, and this will benefit society and future generations. We will give you money to pay for your travel expenses to the clinic and 5 dollars per day.

We will not share the identity of participants in the study with anyone. The information that we collect from this study will be kept confidential. Any information collected about you will have a number on it instead of your name. Only the study team members will know what your number is, and we will lock that information up.

We will share the knowledge that we get from this study with you before it is made available to the public. Confidential information will not be shared. There will be small meetings in the community, and these will be announced. Afterwards, we will publish the results and make them available so that other interested people may learn from our study.

This proposal has been reviewed and approved by Ethical Committee, Department of Medical Research (Lower Myanmar), which is a committee whose task it is to make sure that study participants are protected from harm. If you wish to find about more about the IRB, contact The Secretary, Ethical Committee, Department of Medical Research, No 5, Ziwaka Road, Yangon 11191, Myanmar; Telephone Number 01 375457 Extension 276.

**Part II. Certificate of consent**

I have been invited to participate in a study of a medicine used to treat malaria.

I have read the above information, or it has been read to me. I have had the opportunity to ask questions, and any questions that I have asked have been answered to my satisfaction. I consent voluntarily to participate in this study.

| Print name of participant: |  |  |
| --- | --- | --- |
| Signature of participant: |  |  |
| Date: |  |  |
|  |  | (dd/mmm/yyyy) |

**Witness’ signature:** (A witness’ signature and the patient’s thumbprint are required only if the patient is illiterate. In this case, a literate witness must sign. If possible, this person should be selected by the participant and should have no connection with the study team.)

I have witnessed the accurate reading of the consent form to the potential participant, who has had the opportunity to ask questions. I confirm that the participant has given consent freely.

| Print name of witness: |  |  |  | and thumbprint of participant: |
| --- | --- | --- | --- | --- |
| Signature of witness: |  |  |  |  |
| Date: |  |  |  |  |
|  |  | (dd/mmm/yyyy) |  |  |

**Investigator’s signature:**

**I have accurately read or witnessed the accurate reading of the consent form to the potential participant, who has had the opportunity to ask questions. I confirm that the participant has given consent freely.**

| Print name of investigator: |  |  |
| --- | --- | --- |
| Signature of investigator: |  |  |
| Date: |  |  |
|  |  | (dd/mmm/yyyy) |

A copy of this informed consent form has been provided to the participant. _____ (initials of the principal investigator or assistant).

**Example of a consent statement for a pregnancy test**

I have been invited to participate in a study on the medicine used to treat malaria. I have been asked to supply a specimen of urine at the first visit and at day 28 or on the day of withdrawal from the study, all of which will be used for pregnancy testing. I understand that the results of the tests will be kept fully confidential and anonymous. I understand that I must avoid becoming pregnant during the study because the medicine I will be taking would be dangerous for my child. I have discussed the different methods of birth control with my doctor, and I have been offered condoms. I understand that if the test is positive, I will not be eligible to participate in this study.

**Participant’s signature:**

I accept to be tested. _____ (participant’s initials) or

I do not want to be tested, and I have not signed the consent form below. _____ (participant’s initials)

| Print name of participant: |  |  |
| --- | --- | --- |
| Signature of participant: |  |  |
| Date: |  |  |
|  |  | (dd/mmm/yyyy) |

**Witness’ signature:** (A witness’ signature and the thumbprint of the participant are required only if the participant is illiterate. In this case, a literate witness must sign. If possible, this person should be selected by the participant and should have no connection with the study team.)

I have witnessed the accurate reading of the consent form to the potential participant, who has had the opportunity to ask questions. I confirm that the participant has given consent freely.

| Print name of witness: |  |  |  | and thumbprint of the participant: |
| --- | --- | --- | --- | --- |
| Signature of witness: |  |  |  |  |
| Date: |  |  |  |  |
|  |  | (dd/mmm/yyyy) |  |  |

**Investigator’s signature:**

I have accurately read or witnessed the accurate reading of the consent form to the potential participant, who has had the opportunity to ask questions. I confirm that the participant has given consent freely.

| Print name of investigator: |  |  |
| --- | --- | --- |
| Signature of investigator: |  |  |
| Date: |  |  |
|  |  | (dd/mmm/yyyy) |

A copy of this consent statement has been provided to participant. _____ (initials of the principal investigator or assistant).

1. WHO. *Susceptibility of* Plasmodium falciparum *to antimalarial drugs. Report on global monitoring 1996–2004*. Geneva, World Health Organization, 2005 (WHO/HTM/MAL/2005.110) (<http://www.who.int/malaria/resistance>). [↑](#footnote-ref-2)
2. WHO. *Methods and techniques for clinical trials on antimalarial drug efficacy: genotyping to identify parasite populations.* Geneva, World Health Organization, 2008 (<http://www.who.int/malaria/resistance>). [↑](#footnote-ref-3)
3. WHO/GMP. *Standardized data entry for therapeutic efficacy tests*. Geneva, World Health Organization (<http://www.who.int/malaria/resistance>). [↑](#footnote-ref-4)
4. World Health Organization. Severe falciparum malaria. *Transactions of the Royal Society of Tropical Medicine and Hygiene*, 2000, 94(Suppl. 1):1–90. [↑](#footnote-ref-5)
5. WHO. *Susceptibility of* Plasmodium falciparum *to antimalarial drugs. Report on global monitoring 1996–2004*. Geneva, World Health Organization, 2005 (WHO/HTM/MAL/2005.110) (<http://www.who.int/malaria/resistance>). [↑](#footnote-ref-6)
6. http://www.who.int/rpc/research_ethics/en/ [↑](#footnote-ref-7)
